# Responses of transcriptome and metabolome in peanut leaves to dibutyl phthalate during whole growth period

**DOI:** 10.3389/fpls.2024.1448971

**Published:** 2024-09-20

**Authors:** Lixia Fan, Bingchun Zhang, Mingxiao Ning, Shuangjun Quan, Changying Guo, Kai Cui, Lu Chen, Mengmeng Yan, Xianfeng Ren

**Affiliations:** ^1^ Institute of Quality Standard and Testing Technology for Agro-products, Shandong Academy of Agricultural Sciences, Jinan, China; ^2^ Shandong Provincial Key Laboratory of Test Technology on Food Quality and Safety, Jinan, China; ^3^ Shandong Baolai-leelai Bio-tech. Co., Ltd, Taian, China

**Keywords:** peanut, plasticizer pollution, dibutyl phthalate, transcriptome, metabolome

## Abstract

**Introduction:**

The application of agricultural film mulching technology has significantly contributed to increasing crop yield and income, but the pollution caused by residual film has seriously affected agricultural production and the natural environment. Agricultural film is commonly employed to enhance the yield of peanuts; its use may lead to excessive dibutyl phthalate (DBP) residues in peanut kernels. But, limited investigations have been conducted on the regulatory mechanism of peanut leaves in response to DBP exposure throughout the entire growth period.

**Methods:**

To bridge this knowledge gap, we investigated the differences in transcriptome and metabolome of peanut leaves under DBP stress.

**Results:**

According to visual observations, the results of morphological response showed that the growth of peanut plants was significantly inhibited from seedling to pod stage under DBP treatment. Transcriptomic analysis results showed that the genes AH19G05510 (LRR receptor-like serine threonine-protein kinase) and AH20G31870 (disease resistance), belonging to the FAR1 family and bZIP family respectively, may be key genes involved in the resistance to DBP stress throughout its growth stages. Metabolomic analysis results showed that during the initial stage of DBP stress, the key metabolites in peanut leaves response to stress were carboxylic acids and derivatives, as well as fatty acyls. As peanut growth progressed, flavonoids gradually became more prominent in the resistance to DBP stress. By integrating metabolomics and transcriptomics analysis, we have identified that purine metabolism during seedling and flowering stages, as well as the flavone and flavonol biosynthesis pathways during pod and maturity stages, played a crucial role in response to DBP stress.

**Discussion:**

These findings not only provide valuable key gene and metabolic information for studying anti-plasticizer pollution throughout the entire growth period of peanuts, but also offer reference for enhancing crop resistance to plasticizer pollution through genetic modification and metabolic regulation.

## Graphical Abstract

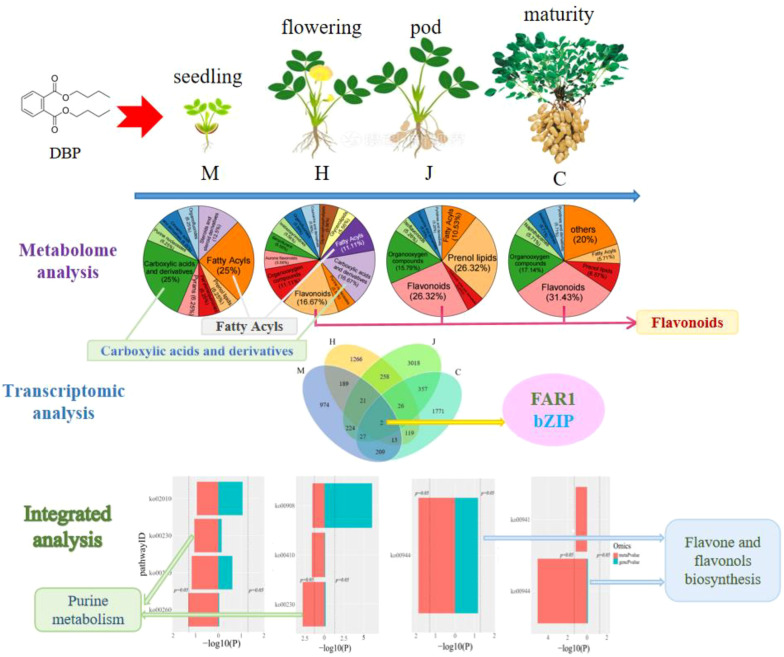

## Highlights

- The key metabolites response to DBP stress were carboxylic acids and derivatives as well as fatty acyls in the initial stage, and flavonoids throughout the later stages of peanut growth.
- AH19G05510 and *AH20G31870*, belonging to *FAR1* and *bZIP* family, may be key genes involved in the peanut’s resistance to DBP stress.
- Purine metabolism, flavone and flavonol biosynthesis pathways plays a crucial role in response to DBP stress.

## Backgrounds

Agricultural film technology has demonstrated positive effects in elevating temperature, conserving soil moisture, and controlling weeds (Lament, 2017). It has evolved into an indispensable agronomic practice in agricultural production, making significant contributions to enhancing crop yield and income. However, prolonged heavy usage and inadequate recycling have led to a gradual accumulation of residual film in the soil. This residual film pollution poses a serious threat to both agricultural production and the natural environment, emerging as a pressing issue that impacts the sustainable development of agriculture (Zhang et al., 2021).

Dibutyl phthalate (DBP) is one of the commonly used phthalate esters (PAEs), which is often used as a plasticizer in agricultural film. It is connected to the plastic polymer by hydrogen bonding or van der Waals forces, resulting in its easy diffusion into the surrounding environment (Yao et al., 2022), so it is a common and frequently detected pollutant in the environment. It is an environmental endocrine disruptor, and has carcinogenicity, teratogenicity and mutagenicity (Sun et al., 2021; Dong et al., 2021), therefore it is recognized as one of the priority environmental pollutants to control in many countries (Lei et al., 2021; Wang et al., 2020). The use of agricultural film is the main source of DBP input in agricultural soil (Zhu et al., 2020; Zhao et al., 2016). In China, DBP concentrations in agricultural soils range from ND (below the detection limit) to 57.7 mg/kg (Lv et al., 2018; Wang et al., 2013). Greenhouse soils with plastic film usage can have residual DBP levels as high as 29.4 mg/kg (Xu et al., 2008).

Under the stress of DBP, crops show adaptive changes at multiple levels, including phenotype, physiological metabolism, and even the microscopic level, which ultimately affect plant growth. Research has shown that DBP negatively affects seed germination in pakchoi (Yao et al., 2022), mung bean (Liu et al., 2014), rape (Ma et al., 2013) and cotton (Lv et al., 2023). It significantly inhibits seedling growth in crops such as broccoli, pepper, radish and spinach (An et al., 1999). On the one hand, DBP can inhibit cell division and slow the growth of crops such as *Arabidopsis thaliana* (Wang et al., 2003), *Scenedesmus obliquus* (Kuang et al., 2003) and cucumber (Zhang et al., 2015b); On the other hand, DBP damages the chloroplast structure and reduces the chlorophyll content, thus affecting the photosynthesis of plants, for example, DBP inhibits the photosynthesis of cotton (Lv et al., 2023), cabbage (Zhao et al., 2016), mung bean (Ma et al., 2014) and wheat (Gao et al., 2017), inducing oxidative damage and ultimately leading to the reduction of biomass. The impact of DBP extends root physiology by causing structural damage of lettuce (Dong et al., 2021) and cucumber (Zhang et al., 2015c) root cells, as well as damaging the spindles on radicle of broad bean (Lai et al., 2014). In terms of crop quality, DBP adversely affected organic acid, vitamin C, soluble protein and soluble sugar levels in cucumber fruit (Wang et al., 2016a), while also decreasing vitamin C and capsaicin levels in chili peppers (Yin et al., 2003). In addition, DBP disrupts gene expression, exhibits genotoxic effects on cotton and broad beans, and up-regulates metacaspase gene expression in wheat, resulting in programmed cell death. Additionally, DBP influences protein profiles, as evidenced by the extraction of 6 differentially expressed protein spots from DBP-treated Chinese cabbage leaves (Liao et al., 2009).

Peanut (*Arachis hypogaea* L.) is a globally significant oil crop that holds substantial economic value. Employing mulching has become a widely utilized cultivation method in order to enhance peanut production and efficiency (Zheng et al., 2019), so peanuts are at greater risk of exposure to DBP contamination. At present, there is a lack of studies on the toxic mechanism of DBP on the whole growth period of peanut plants. Therefore, the interaction between DBP and peanut plants during the whole growth period needs to be explored by multiple omics technology. Multiple omics technology involves the integration of two or more high-throughput omics technologies to comprehensively analyze biological mechanisms. This approach combines transcriptomics, genomics, proteomics, metabolomics and epigenetics to gain a deeper understanding of biological processes (Kim et al., 2016; Zhang et al., 2015a). The utilization of various omics technology has significantly enhanced the efficiency of studying the plant abiotic stress response process, while also providing a foundation for comprehensively analyzing the molecular mechanisms underlying plant stress response. In particular, the combination of transcriptome and metabolome analysis has emerged as a well-established method in multiply omics studies. This technique is widely used to investigate the response mechanisms of plants to different stresses. For instance, several studies have explored the effects of cold stress on the transcriptome and metabolome of various crops such as peanut (Wang et al., 2021a), tobacco (Jin et al., 2017), wheat (Zhao et al., 2019), rapeseed (Jian et al., 2020) and common bean (Yang et al., 2023). The molecular mechanism underlying the impact of salt stress on cotton (Han et al., 2023), peanut (Cui et al., 2018), oat (Xu et al., 2021) and rice (Wang et al., 2021b) was investigated. Additionally, the differential expression genes and differential accumulation metabolites of barley under salt-alkali stress were studied, and the biosynthetic pathway of phenylpropanol has been found, thus establishing the mechanism of salt tolerance (Ho et al., 2020).

The majority of research on DBP stress primarily focuses on the physiological and biochemical changes as well as the toxic effects of crops at specific time points, lacking comprehensive studies throughout the entire growth stage. A comprehensive analysis of metabolomics and transcriptomics represents a potent approach for elucidating the potential toxic mechanism of DBP stress. In this study, the key regulatory factors and metabolic pathways affected by DBP in peanut leaves throughout the entire growth stage were systematically analyzed, which provided valuable insights into understanding how peanuts respond to DBP stress over their complete growth cycle, and laid a foundation for enhancing crop resistance to plasticizer pollution through genetic modification.

## Materials and methods

### Plant material and treatment

The peanut variety Huayu No. 22 was used in this study. Huayu No.22 is an early-maturing, large-seeded peanut variety and the dominant cultivar in Shandong Province, China. It is extensively cultivated in China and serves as the primary export peanut due to its high quality, garnering recognition in both domestic and international markets. The ceramic pots measuring 300 mm × 300 mm (inner diameter × height) was used as the culture device. The experiment was conducted at an experimental field of the Shandong Academy of Agricultural Sciences (Jinan, China) where the GPS coordinates is 36°39′N, 117°04′E). The test areas belong to the typical temperate monsoon climate, the average temperature and average sunshine duration were 25.7 °C and 7.8 h from 0 to 130 days after peanut sowing, respectively. The agricultural soil is loam and the nutrient composition was as follows: pH 5.8, organic matter 14.0 g·kg^-1^, available nitrogen 79.3 mg·kg^-1^, available phosphorus 26.4 mg·kg^-1^ and available potassium 274.7 mg·kg^-1^. The original soil contains 30.8 μg·kg^-1^ DBP, but no other PAEs were detected. The soil used in the experiment consisted of two treatments: the one without DBP supplement as the control (CK), and the other with additional supplement of DBP to a final concentration approximately of 62 mg·kg^-1^. The fertilization rate applied was N (urea, CH_4_N_2_O) 200 mg·kg^-1^, P [(CaPO_4_)_2_·H_2_O] 150 mg·kg^-1^, K (K_2_SO_4_) 200 mg·kg^-1^. Each pot was filled with 13 kg of the soil. Each treatment group consisted of 5 pots. Soil water content adjusted to 16%, and the soil was allowed to age for 14 days prior to sowing. Full seeds of uniform size were selected and evenly distributed in each pot, with 3 holes made in each pot and 2 seeds placed in each hole. This ensured that each hole had a strong seedling after emergence. Regular and quantifiable watering was carried out throughout the experiment.

### Transcriptome sequencing

Leaves were collected from the same position on the peanut main stem at seedling, flowering, pod, and maturity stages. Transcriptome determination was conducted on three biological replicates. The total RNA was extracted and RNA quality was assessed by OD value and the integrity number (RIN) (Kvastad et al., 2021; Ma et al., 2022). The initial RNA for library construction was total RNA, with the total amount >1000 ng. Using oligo(dT) beads enriched mRNA with polyA tail, then the claved mRNA was synthesized into cDNA using random primers. After terminal repair, A-tail was added, double-stranded cDNA was connected to sequencing joints, and about 200bp cDNA was screened by AM Pure XP beads. PCR amplification and purification of PCR products using AM Pure XPbeads were performed to obtain libraries (Mo et al., 2022).

The fastp 0.23.2 software was employed for data quality control filtering. The filtered data were then utilized for gene expression analysis, differential gene screening, and bioinformatics analysis. The reference genome used for mapping the clean reads was the genome of the peanut cultivar Shitouqi (PGR: http://peanutgr.fafu.edu.cn/Download.php). The expression values FPKM (Fragments per kilobase per million) of all genes in a sample were calculated. DESeq2 1.20 was employed for DEGs analysis, with the filter condition for differentially expressed multiples set at |log_2_ (FoldChange)| > 1.0 and a significance *p-*value ≤ 0.05 (Hou et al., 2023; Ma et al., 2022; Zhang et al., 2022).

GO functional enrichment analysis and KEGG pathway enrichment analysis were performed by clusterProfiler 4.2.0 software. GO consists of biological process, cellular component, and molecular function. Significance threshold for GO functional and KEGG pathways enrichment were both set at *p*adj < 0.05.

### Metabolite extraction and profiling

Leaves were collected from the same position on the peanut main stem at seedling, flowering, pod, and maturity stages. Each treatment had five biological replicates. The leaves were quickly placed in liquid nitrogen and stored at -80°C for metabolome analysis. The leaves were ground to a powder in liquid nitrogen. Appropriate samples were taken and mixed with 1 ml of a water: acetonitrile: isopropyl alcohol solution in a 1:1:1 ratio (v/v). The mixture was vortexed for 60 seconds, sonicated at low temperature for 30 minutes, and then centrifuged at 12,000 rpm at 4°C for 10 minutes to obtain the supernatant. The supernatant was then placed at -20°C for 1 hour to precipitate proteins. After centrifugation at 12,000 rpm at 4°C for 10 minutes, the supernatant was collected (Yuan et al., 2021). The sample was then vacuum dried and reconstituted in 200 μL of 50% ACN. The mixture was vortexed and centrifuged at 14,000 rmp and 4°C for 15 minutes. The supernatant was obtained and subjected to qualitative and quantitative analysis of metabolites using ultra-high-performance liquid chromatography with tandem electrostatic field orbitrap mass spectrometry (UHPLC-Q Exactive HF-X, Thermo, USA). The chromatographic column was Waters HSS T3 (100*2.1 mm, 1.8μm) and the column temperature was 40°C. The injection volume was 2μl and the flow rate was 0.3 ml/min (Li et al., 2022). The solvent systems contained mobile phase A (0.1% formic acid-aqueous solution) and phase B (0.1% formic acid-acetonitrile). Student’s t-test combined with multivariate analysis of OPLS-DA was used to identify DAMs between CK and DBP treated at the four time points, a threshold of VIP (Variable Importance for the Projection) score>1 and *p-* value<0.05 were used (Yang et al., 2022).

### Combined analysis of transcriptomic and metabolomics results

The intrinsic correlation between metabolome and transcriptome data was evaluated using O2PLS method. Initially, transcriptional and metabolic models were constructed, and the scores for each sample were calculated to generate the joint score map. The loading values for each gene and metabolite were also calculated to create the loading map. The joint score plot displays the relationship between the two data matrices, and metabolites/genes with high loading values are considered crucial for the similarity of the two data sets. Finally, the top 15 DAMs/DEGs with an absolute value of load value were selected to generate the bar chart. Correlation network analysis was conducted based on Pearson correlation coefficient. The threshold of correlation coefficient in this study was ±0.8, and the correlation significance was *p*<0.05.

## Results

### Transcriptomic analysis of peanut leaves exposed to DBP stress

#### General description of transcriptome data

The transcriptomic changes in peanuts under CK and DBP treated groups were compared at four different growth stages. The RIN value of the isolated RNA were more than 7 (**Supplementary Table S1**) (Pandey et al., 2022). Through transcriptome sequencing, a total of 38,055,756-126,187,214 clean reads were obtained from all the samples. The quality of the reads was assessed based on Q20 and Q30 bases, with both exceeding 98.67% and 95.43% respectively. The Guanine and Cytosine (GC) content of the reads ranged from 44.58% to 57.88% (**Supplementary Table S1**), indicating high-quality sequencing data. The mapping percentages of the clean reads to the reference genome ranged from 92.11% to 97.24%.

**Figure 1:**
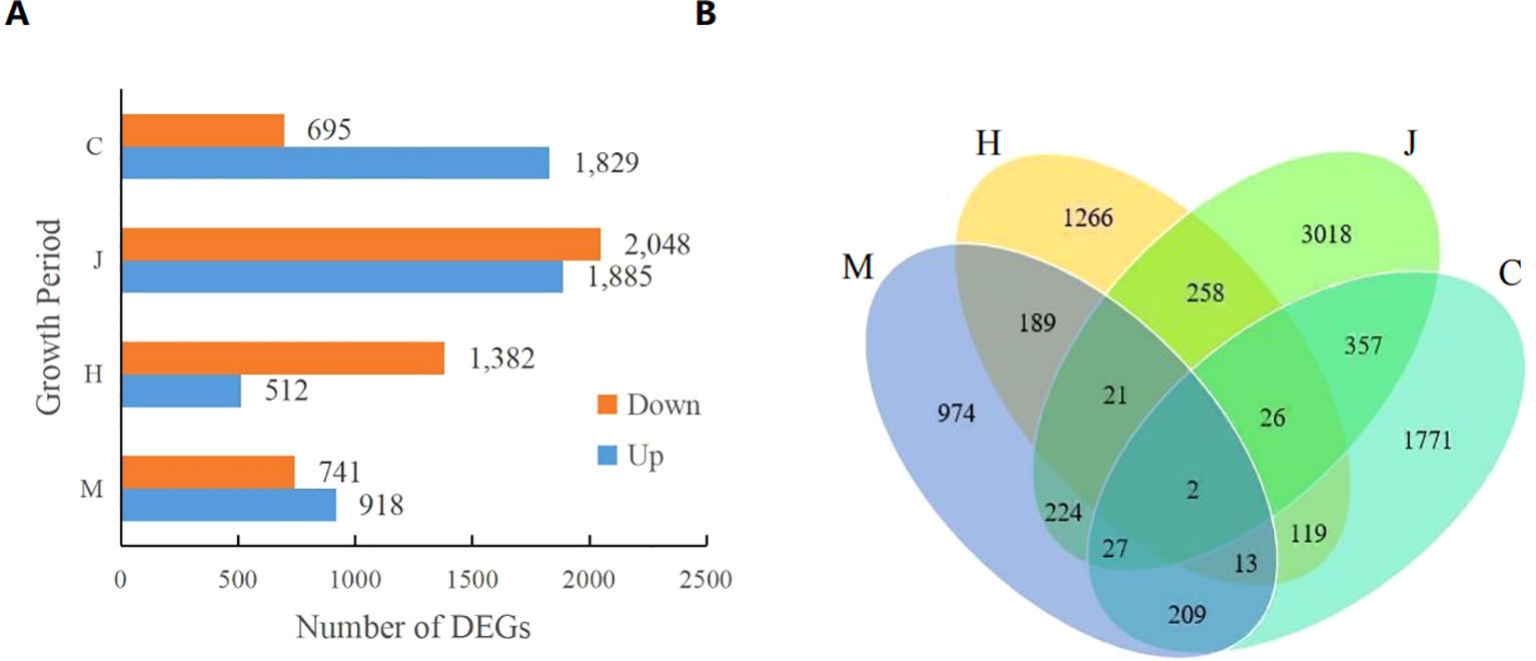
Number of DEGs. **(A)** shows the upregulation and downregulation of DEGS in different comparison groups and **(B)** shows the Venn plot of DEGS.

As shown in **Figure 1A**, a total of 1659 differential expression genes (DEGs) (741 up-regulated and 918 down-regulated), 1894 DEGs (1382 up-regulated and 512 down-regulated), 3933 DEGs (2048 up-regulated and 1885 down-regulated), and 2524 DEGs (695 up-regulated and 1829 down-regulated) were identified at the seedling, flowering, pod and maturity stages, respectively. The number of DEGs gradually increased during the seedling, flowering, and pod stages, but decreased at the mature stage. This indicates that DBP stress initially causes significant changes in the peanut transcriptome at different growth stages, with the effect diminishing at later stages. Furthermore, Venn diagram analysis revealed that there were two overlapping DEGs across the four growth stages of peanut (**Figure 1B**). These genes are identified as AH19G05510 (LRR receptor-like serine threonine-protein kinase, FAR1 family) and AH20G31870 (disease resistance, bZIP family).

### GO functional classification of DEGs

GO enrichment analysis was performed on the DEGs from peanuts leaves at different growth stages (**Supplementary Figure S1**). The results revealed that a total of 669, 2990, 1590 and 4257 GO entries enriched for the DEGs at seedling, flowering, pod and maturity stages, respectively.

At the seedling stage, the top enriched GO terms in the cellular component category were related to “cell wall” and “external encapsulating structure”. In terms of the biological process category, the most significantly enriched terms were “pollination” and “multi-multicellular organism process”. For the molecular function category, “carbon-oxygen lyase activity” and “ATPase-coupled transmembrane transporter activity” were the most frequently annotated entries for the DEGs.

At the flowering stage, the most annotated entry for DEGs in terms of cellular component was the “supramolecular complex”. In biological processes, the most annotated entry for DEGs was “response to auxin”. In terms of molecular function, “channel activity” and “passive transmembrane transporter activity” were the most annotated entries for DEGs.

At the pod stage, the most annotated entry for DEGs in terms of cellular component was the “external encapsulating structure”. In biological processes, “carbohydrate biosynthetic process” was the most annotated entry for DEGs. In terms of molecular function, the most annotated entry for DEGs was “oxidoreductase activity, acting on CH-OH group of donors”.

At the mature stage, the most annotated entries for DEGs in terms of cellular component were the “chloroplast thylakoid membrane” and “plastid thylakoid membrane”. “Chlorophyll binding” was identified as the most annotated entry of DEGs in the biological process category. This finding is consistent with previous research, which demonstrated that DBP can influence the chlorophyll content in wheat and Chinese flowering cabbage, resulting in a decline in photosynthetic capacity (Zhao et al., 2016). Additionally, the DEGs related to the molecular function category showed that “photosystem” was the most annotated entry.

Furthermore, the analysis of DEGs in peanut leaves at seedling, flowering and pod stages revealed that a significant number of genes associated with the “cell wall” and “external encapsulating structure” were identified. This may be attributed to the infiltration of exogenous substances. Strengthening the cell walls is a typical response in biological processes when plants encounter stressful situations, as it enhances their resilience (Duan et al., 2016). These findings imply that the active recombination or modification of the cell wall may be a common mechanism employed by peanut plants to counteract the toxicity caused by DBP contamination at different growth stages.

**Figure 2:**
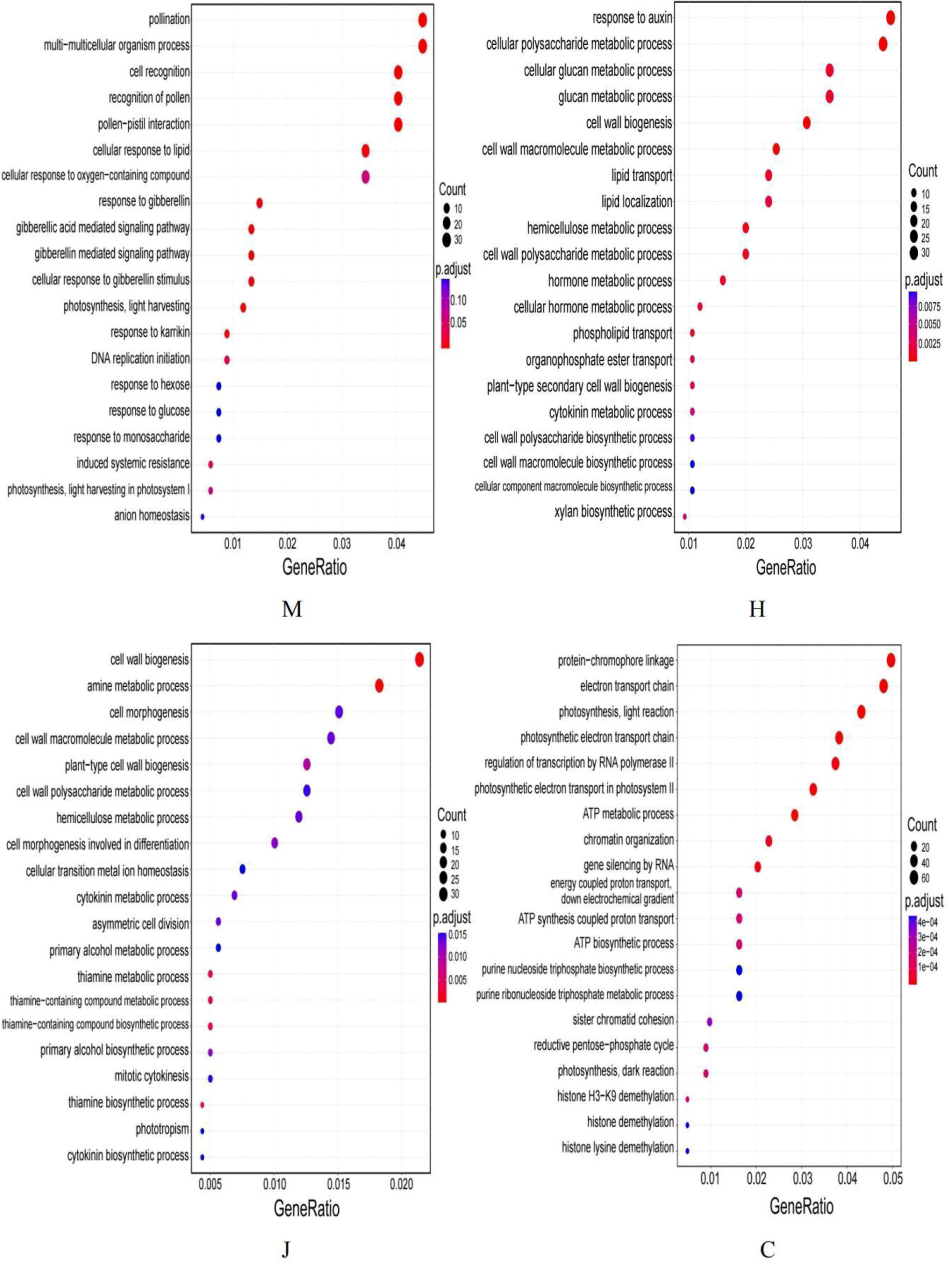
GO enrichment analysis.

The scatter plot (**Figure 2**) was generated based on the most significant 20 terms identified from the GO enrichment analysis results. At the seedling stage, 30 genes were found to be enriched in pollination and multi-multicellular organism process. During the flowering stage, 34 genes were enriched in response to auxin. At the pod stage, 34 genes were associated with cell wall biogenesis. At the mature stage, 61 genes were enriched in protein-chromophore linkage. Furthermore, the analysis of the directed acyclic graph (**Supplementary Figure S2**) revealed that the top 5 top GO terms in all four growth periods of peanut belonged to the broad category of “biological processes”. Following treatment with DBP, DEGs enriched in the “multi-multicellular organism process” at the seedling stage were found to potentially regulate the “recognition of pollen”. The DEGs at the flowering stage were primarily involved in “cellular polysaccharide metabolic processes”, “cell wall macromolecule metabolic processes”, “cell wall biogenesis” and “response to auxin”. The DEGs at the pod stage were mainly enriched in “amine metabolic processes”. On the other hand, the DEGs at the maturity stage were mainly enriched in “protein-chromophore linkage”. Moreover, the DEGs enriched in the electron transport chain could modulate the “photosynthetic electron transport in photosystem II” by regulating the “photosynthetic electron transport chain”. Furthermore, significant enrichment of DEGs was observed in various GO function associated with peanut growth under DBP stress at different stages.

### KEGG pathway functional analysis of DEGs

**Figure 3:**
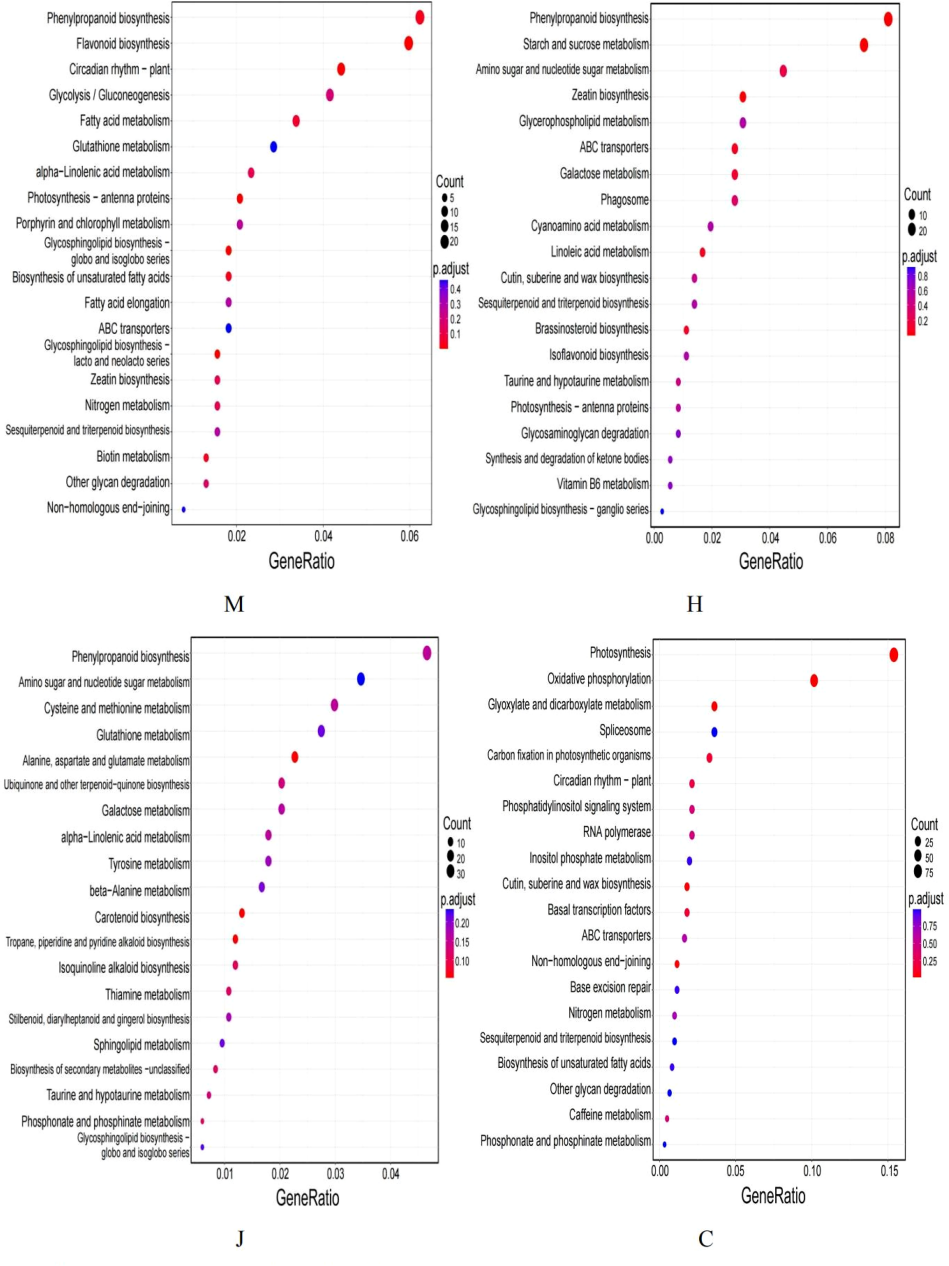
The top 20 KEGG scatterplots under DBP stress.

The Kyoto Encyclopedia of Genes and Genomes (KEGG) pathway enrichment analysis of DEGs (**Figure 3**) revealed the most enriched pathways at the seedling stage to be phenylpropanoid biosynthesis, flavonoid biosynthesis, circadian rhythm-plant, glycolysis/gluconeogenesis, endocytosis, starch and sucrose metabolism, among others. At the flowering stage, the enriched metabolic pathways included phenylpropanoid biosynthesis, starch and sucrose metabolism, amino sugar and nucleotide sugar metabolism, zeatin biosynthesis, glycerol phosphatelipid metabolism and others. At the pod stage, the enriched metabolic pathways primarily included phenylpropanoid biosynthesis, amino sugar and nucleotide sugar metabolism, cysteine and methionine metabolism, glutathione metabolism and others. At the mature stage, we found that the DEGs were primarily enriched in several metabolic pathways, including photosynthesis, oxidative phosphorylation, glyoxylate and dicarboxylic acid metabolism and spliceosome, carbon fixation of photosynthetic organisms and phenylpropanoid biosynthesis.

Interestingly, we also observed that the phenylpropanoid biosynthesis pathway was consistently enriched at the seedling, flowering, pod and maturity stages. Phenylpropanoid compounds have been known to play a critical role in plant growth and stress response (Wu et al., 2022). This finding suggests that the phenylpropanoid biosynthesis pathway is crucial throughout the different stages of peanut growth under DBP stress. Additionally, we found that the starch and sucrose metabolic pathways were commonly enriched at the seedling, flowering and pod stages. Moreover, at the flowering and pod stages, the DEGs were also enriched in the amino sugar and nucleotide sugar metabolism pathways. Lastly, the endocytosis pathway was found to be enriched at the seedling and pod stages. Overall, our results indicate that these common metabolic pathways are important for peanut growth and development under DBP stress, acting as key players in various biological processes, such as energy production, stress response and nutrient metabolism.

### Metabolome profiling of peanut leaves in response to DBP

**Figure 4:**
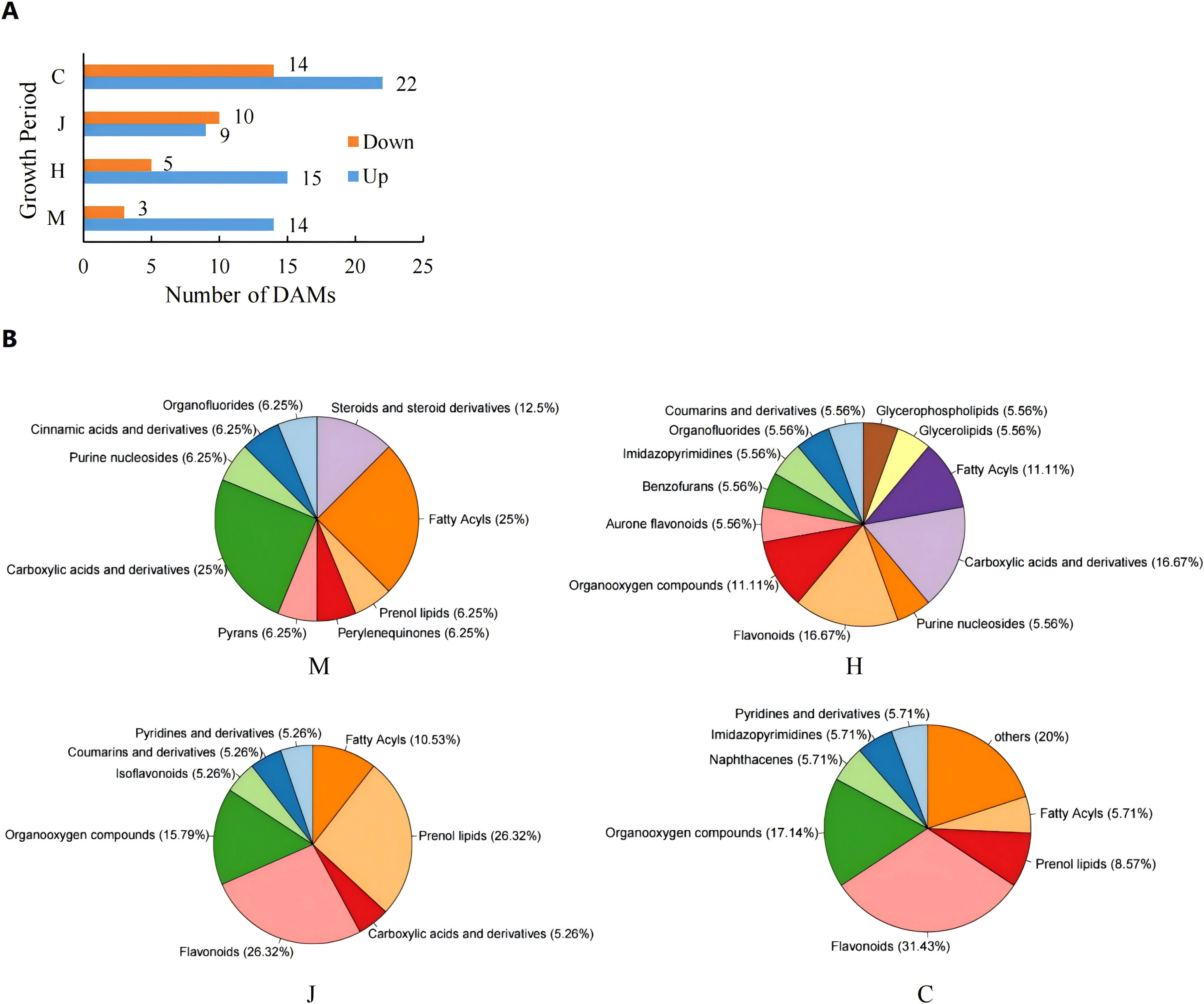
Comparison of metabolite leveis under DBP stress. **(A)** Number of DAMs after treatment with DBP. **(B)** Classification of DAMS.

To investigate the role of metabolism in the physiological changes of peanuts during DBP stress, we collected leaves at four different stages of peanut growth (with five biological replicates) and analyzed their metabolic changes using UPLC-MS/MS. Our results indicated the detection of a total of 997 metabolites. Among these metabolites, 17, 20, 19, and 36 differential accumulation metabolites (DAMs) exhibited significantly different response levels to DBP stress compared to CK at the seedling, flowering, pod, and mature stages, respectively (**Figure 4A**). Specifically, 14, 15, 9, and 22 DAMs were up-regulated, respectively. The classification of DAMs at different stages was depicted using a pie chart (**Figure 4B**). As depicted in the chart, carboxylic acids and derivatives, fatty acyls were the most abundant at the seedling stage, accounting for 25% each. At the flowering stage, carboxylic acids and derivatives, flavonoids were the most abundant, constituting 16.67% each. The abundances of flavonoids and prenol lipids were the highest at the pod stage, accounting for 26.32% each. At the mature stage, the highest abundance was found in flavonoids, accounting for 31.43%. The metabolite profiles of the four stages were well separated according to orthogonal partial least squares-discriminant analysis (OPLS-DA) (**Supplementary Figure S3**). The DBP-treated and CK samples were separated, indicating that DBP had significant effects on the accumulation patterns throughout peanut’s growth period. Similarly, the metabolic changes of different metabolites in peanut leaves treated with DBP were significantly distinct from those in CK. Clustering analysis showed significant separation between DBP-treated and CK at the seedling and maturity stages (**Supplementary Figure S4**). At the flowering stage and pod stage, there was only one sample of the DBP treatment that was grouped into the same category as the CK, possibly due to individual differences. Generally, there was significant cluster separation of peanut leaves between the DBP treatment and CK groups in each period. The proportion of DAMs with high expression gradually decreased from the seedling stage to the pod stage, and slightly increased at the maturity stage, which was speculated to be the result of peanut gradually adapted to the stressed environment. As shown in **Supplementary Figure S5**, KEGG pathway analysis revealed that at the seedling stage, the DAMs were mainly enriched in caprolactam degradation, glycine, serine and threonine metabolism, phenylalanine metabolism, purine metabolism and steroid hormone biosynthesis. At the flowering stage, the main enriched biosynthesis pathways were purine metabolism, zeatin biosynthesis, pantothenate and CoA biosynthesis, and beta-Alanine metabolism. Specifically, flavone and flavonol biosynthesis was enriched at the pod stage, while flavonol biosynthesis, caprolactam degradation, flavone and flavonol biosynthesis were enriched at the maturity stage. These findings suggest that the metabolic pathways of DAMs significant enrichment were closely related to DBP stress at different growth stages.

### Integrated analysis of the metabolome and transcriptome

**Figure 5:**
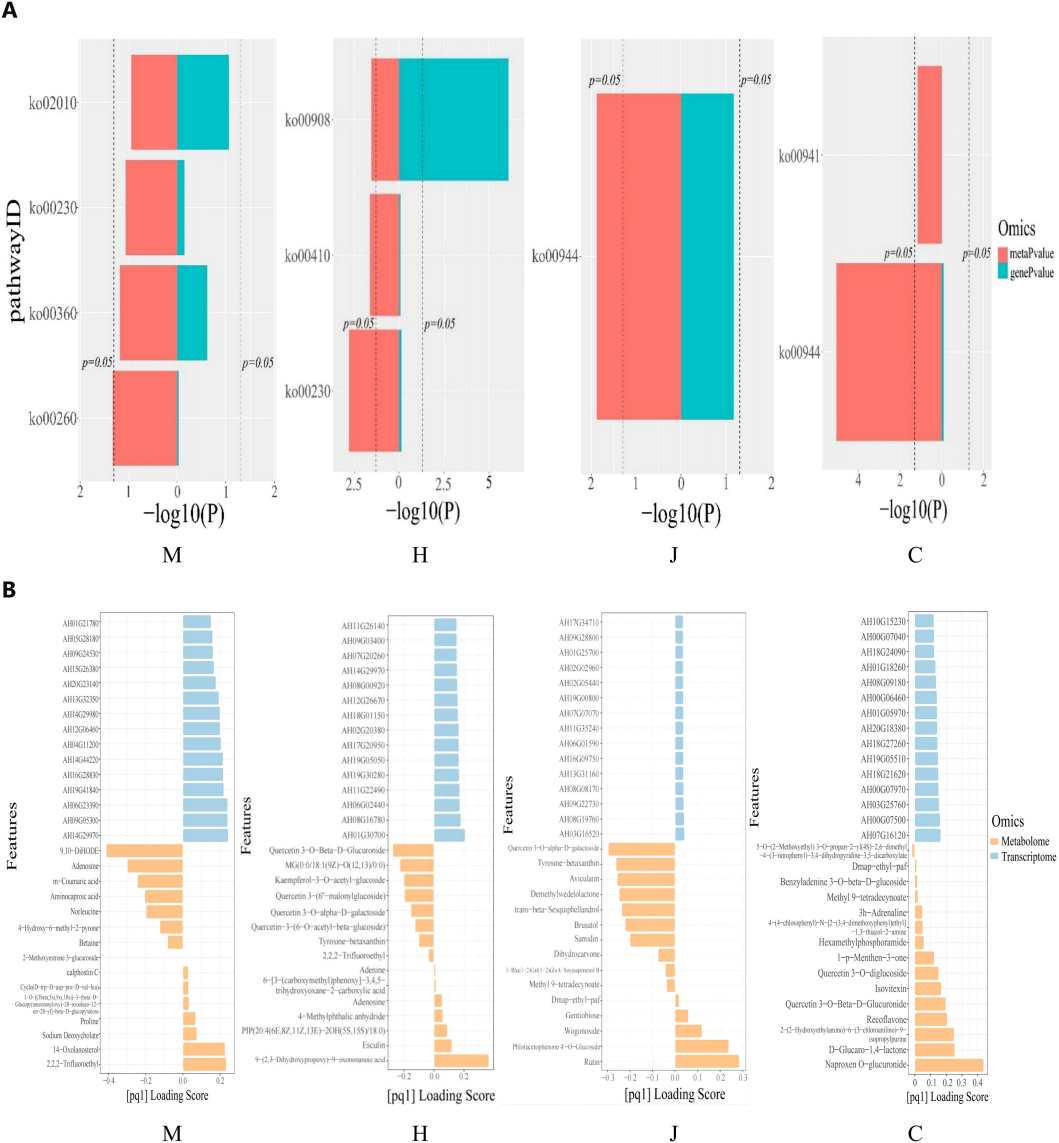
Correlation analysis between meusbolites and transcripts. **(A)** Histograns of the KEGG pathway crunched by the DEGs and DAMS. **(B)** The top15 DEGs and DAMs loading histogram.

Through pathway co-enrichment analysis of genes and metabolites, we identified four, three, one and two co-enriched metabolic pathways at the seedling, flowering, pod and maturity stages, respectively (**Figure 5A**). At the seedling stage, the enriched metabolic pathways included glycine, serine and threonine metabolism; phenylalanine metabolism; purine metabolism; and ABC transporter. Purine metabolism, β-alanine metabolism and zeatin biosynthesis were enriched at the flowering stage. The pod stage was characterized by the enrichment of flavone and flavonols biosynthesis. Finally, the mature stage showed enrichment of flavone and flavonols biosynthesis, as well as flavonoid biosynthesis. The differential genes with *p*-value>0.05 were glycine, serine and threonine metabolism at the seedling stage and purine metabolism, β-alanine metabolism and zeatin biosynthesis at the flowering stage. Flavone and flavonols biosynthesis was observed during both the pod and mature stages of peanut. Notably, zeatin biosynthesis at flowering stage showed *p*-value>0.05. Furthermore, purine metabolism was found to be enriched during both the seedling and flowering stages. The enrichment of flavone and flavonol biosynthesis was consistent with the common characteristics of peanut in response to DBP stress during both the pod and mature stages.

Gene/metabolite two-way orthogonal partial least squares (O2PLS) loading map (**Supplementary Figure S6**) and the top 15 DEGs/DAMs bar chart (**Figure 5B**) shows DEGs and DAMs with absolute Top15 load values. They are of great significance for us to search for genes regulating metabolites in transcriptomics.

Plant adaptation to abiotic stress involves the interaction of multiple physiological pathways, forming a complex regulatory network. To identify the correlation between DEGs and DAMs at key regulatory positions, a correlation network diagram (**Supplementary Figure S7**) was constructed for the DEGs and DAMs at different growth stages of peanut. The results were summarized in **Supplementary Table S2**, which provides a summary of the number of DEGs associated with DAMs exhibiting significant trends.

## Discussion

Currently, plant stress has emerged as a prominent research area in plant science, particularly focusing on plant seeds and seedlings, and studies of DBP stress is mainly concentrated in these two stages. However, there is a paucity of studies regarding DBP stress throughout the entire plant growth cycle. In reality, it is more practical to investigate the stress experienced by plants throughout their entire lifespan, as they are immobile organisms and are likely to endure continuous stress. The resistance mechanism of peanut plants to DBP stress throughout their entire growth period has not yet been explored.

### Transcriptome analysis of peanut leaves under DBP stress

Plant responses to stress involve intricate biological processes encompassing physiology, biochemistry and molecular events. Among these processes, plants respond to various stresses by altering the expression patterns of relevant genes. These genes include those encoding proteins with specific functions (such as structural proteins and enzymes) as well as genes involved in regulating downstream gene expression (such as transcription factors). The identified gene IDs in this study were *AH19G05510* (LRR receptor-like serine threonine-protein kinase) and *AH20G31870* (disease resistance), which belong to the *FAR1* family and *bZIP* family, respectively. Current studies on *FAR1* transcription factors mainly focus on their role in regulating plant optical signals. *FAR1* is an important positive regulator of the phytochrome A (*phyA*) signaling pathway, which is involved in the regulation of gene expression by assisting *phyA* to enter the nucleus through the identification and activation of genes containing *FBS* cis-acting elements (Lin et al., 2007; Ouyang et al., 2011). Some studies have demonstrated that overexpression of *FAR1* transcription factors can significantly enhance drought resistance in *Arabidopsis* and peanut (Yan et al., 2020). *FAR1* is a class of transcription factors derived from transposase, and it directly activates the expression of *FHY1/FHL* genes in response to far-red light. Moreover, *FAR1* plays a crucial regulatory role in plant starch anabolism and energy deficiency caused by carbon deprivation (Casal, 2000; Chen et al., 2012). Light serves as the energy source for plant growth and development. Phytochromes play a crucial role in the photoreceptor network of higher plants. They detect red and far red light and directly or indirectly control downstream transcription factors, thereby regulating gene expression and influencing plant growth and development processes, such as seed germination, seedling de-etiolation and chloroplast movement (Heijde and Ulm, 2012; Furuya, 2003; Tepperman et al., 2001). Leucine-rich repeat receptor-like protein kinases (LRR-RLKs) represent the largest class of receptor protein kinases in plants, playing important roles in plant growth, development, and defense processes (Liu et al., 2017). Reversible protein phosphorylation by protein phosphatases and kinases serves as the central mechanism for plant sensing and response to abiotic stress (Tong et al., 2010). Furthermore, key signaling molecules that play pivotal roles in plant responses to both biotic and abiotic stress are represented by protein kinases. For example, FLAGELLIN-SENSITIVE 2 (FLS2) is implicated in plant defense and pathogen recognition (Gomez and Boller, 2000), while BIR1 and SOBIR1 are involved in regulating cell death and innate immunity in *Arabidopsis* (Gao et al., 2009).

The *bZIP* (basic leucine zipper) (Jakoby et al., 2002) transcription factor family is involved in plant stress response and regulates plant stress resistance. The *bZIP* transcription factor participates in various biological processes, including the regulation of plant growth and development, such as seed germination (Gibalová et al., 2009) growth and development of plant roots, stems and other organs (Van Leene et al., 2016; Ma et al., 2018; Alex, 2022; Yang et al., 2002), plant senescence (Yang et al., 2002; Alex, 2022) and light signal transduction (Chang et al., 2019). Moreover, it is also involved in plant responses to biotic (Chang et al., 2017; Zhang et al., 2015d; Ramakrishna et al., 2018) and abiotic stresses (Hsieh et al., 2010; Wang et al., 2016b), among others. Overexpression (Xiang et al., 2008; Zhang et al., 2011) or silencing the expression (Fitzgerald et al., 2010) can enhance the resistance of plants to biotic and abiotic stresses. According to these reports and our studying results, the genes AH19G05510 (LRR receptor-like serine threonine-protein kinase) and AH20G31870 (disease resistance), belonging to the FAR1 family and bZIP family respectively, may be key genes in peanut leaves involved in the resistance to DBP stress throughout its growth stages.

### Metabolome analysis of peanut leaves under DBP stress

The concomitant alteration in metabolism is the primary response of plants to stress. In other words, plants modulate their metabolic network to elicit a range of specialized metabolites, enabling them to defend against biotic or abiotic stress. Plants have developed specific defense mechanisms to cope with various stresses. Signaling molecules stimulate changes in plant defensive metabolites, which play a crucial role in regulating plant metabolism under stress (Kazan, 2015; Andy, 2016). Analyzing changes in plant metabolites after stress or stimulation provides a comprehensive understanding of the interactions between plants and their environment (Arbona and Gómez-Cadenas, 2016). After DBP stress, at seedling stage, more differential metabolites were promoted in peanut leaves, include 3 amino acids: norleucine, betaine and proline; and 4 fatty acyls species. The following compounds exhibited up-regulation during the flowering period: carboxylic acids and derivatives such as calcium pantothenate, leucylphenylalanine and tyrosine- betaxanthin; flavonoids such as quercetin 3-O-beta-D-glucuronide, quercetin 3-(6”-malonylglucoside) and quercetin 3-O-alpha-D-galactoside. At pod stage, among the flavonoids species, luteolin 7-glucuronide, quercetin 3-O-alpha-D-galactoside, rutin, avicularin were up-regulated, while wogonoside was down-regulated; Prenol lipids species exhibited up-regulation with rothin B, but trans-beta-sesquiphellandrol, brusatol, dihydrocarvone and 3-Rha(1-2)Gal(1-2)GluA-soyasaponenol B were down-regulated. At maturity stage, 10 flavonoids, such as quercetin, isoquercitrin, recoflavone, were found to be up-regulated in this study. Conversely, two down-regulated flavonoids, lonicerin and 5-O-(2-methoxyethyl) 3-O-propan-2-yl(4S) -2,6-dimethyl-4-(3-nitrophenyl)-3, 4-dihydropyridine-3,5- dicarboxylate, were also observed.

Metabolomics is an effective approach to studying plant stress resistance. Amino acids, as the basic units of proteins (Zhao et al., 2014), play essential roles in various biological processes. They not only participate in the synthesis and transformation of ammonia-containing substances such as hormones, but also effectively resist oxidative damage in plants. Betaine (Vermel et al., 2002) and proline, which are osmolytes, are synthesized in large quantities in response to stress. These osmolytes assist in regulating the osmotic pressure within plant cells and stabilize the spatial structure of biological macromolecules, including proteins, thereby ensuring their activity and function (Younis et al., 2009). Additionally, proline acts as an antioxidant within plants, removing excessive reactive oxygen species and reducing the toxicity of contaminants on peanut plants. Phytolipids, on the other hand, play a crucial role in responding to stress, as well as in the growth and development of plants (Chen et al., 2018). Lipids have multiple functions in plant metabolism, serving as a source of carbon and nitrogen. They are also important structural components of cell membranes, forming the lipid bilayer and protective lipids on the plant surface (Chen et al., 2018). Changes in lipid composition reflect alterations in membrane lipid components, which indicate the response of plants to environmental stress (Perlikowski et al., 2016). Therefore, carboxylic acids and derivatives, as well as fatty acyls might be the key metabolites in peanut leaves response to PDB stress during the initial growth stage.

Flavonoids, the most common secondary metabolites, are upregulated and present at different stages of plant development such as the flowering stage, pod stage and maturity stage in our study. They play crucial roles in various aspects of plant biology, including growth and development, enhancement of plant tolerance to biotic and abiotic stresses, and promotion of competitive compounds within the plant population. Flavonoid compounds are vital for enhancing the plant’s competitive ability and disease resistance (Xu et al., 2023). Flavonoids play a crucial role in plant stress resistance as potent antioxidants. They have the ability to regulate factors in the REDOX system, activate the plant immune system, and stimulate the production of enzymes such as peroxidase (POD), superoxide dismutase (SOD) and catalase (CAT) (Taylor and Grotewold, 2005; Cavaiuolo et al., 2013). It was suggested that peanut could improve the stress resistance of host by promoting the production of flavonoids in our study.

### Integrated analysis of the metabolome and transcriptome to peanut leaves under DBP stress

In addition, various metabolic pathways and signal transduction pathways also play crucial roles in the plant’s response to stress. Purine metabolism was found to be enriched during both the seedling and flowering stages. At the flowering stage, the DAMs adenosine and adenine was down-regulated. Adenosine, a purine nucleoside, is involved in various biochemical processes such as energy transfer through ATP or ADP and signaling through cAMP. Both the pod and mature stages of the peanut leaves showed enrichment in the flavone and flavonol biosynthesis pathways. Previous studies have shown that exposure to abiotic stress induces the expression of genes involved in the “flavonoid and flavonol biosynthesis” pathway in *Verbena bonariensis* (Wang et al., 2018) and *Camellia sinensis* (Mutwakil et al., 2017), flavonoids are believed to enhance stress resistance by improving antioxidant capacity. Therefore, purine metabolism during seedling and flowering stages, as well as the flavone and flavonol biosynthesis pathways during pod and maturity stages, played a crucial role in response to DBP stress.

## Conclusion

The impact of DBP stress on peanut growth was significantly negative. The results of our study suggest that AH19G05510 and AH20G31870 which belong to the *FAR1* and *bZIP* families respectively may serve as key genes in peanut resistance to DBP stress throughout its growth stages. Carboxylic acids and derivatives, as well as fatty acyls were the primary metabolites in the initial response of peanut leaves to DBP stress, later transitioning to flavonoids. Purine metabolism, biosynthesis of flavonoids, and flavonols play pivotal roles in peanut’s response to DBP stress at various growth stages. These findings substantially enhance our understanding of the mechanisms underlying peanut’s response to DBP stress at different developmental stages and lay a solid foundation for further exploration into the functions of relevant genes and metabolites in DBP tolerance.

## Data Availability

The data presented in the study are deposited in the NCBI repository, accession number PRJNA1033427.
