# Supplementary material for: Responses of transcriptome and metabolome in peanut leaves to dibutyl phthalate during whole growth period
Source: Front Plant Sci. 2024 Sep 20;15:1448971. doi: 10.3389/fpls.2024.1448971 (PMC11452913; doi:10.3389/fpls.2024.1448971)
Supplement: Supplementary file 1 [file DataSheet1.zip › Figure S5.PDF]

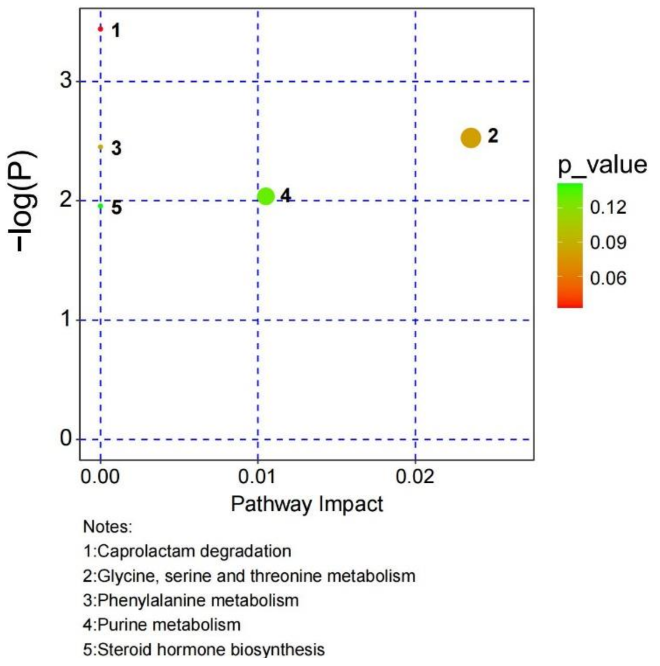

M

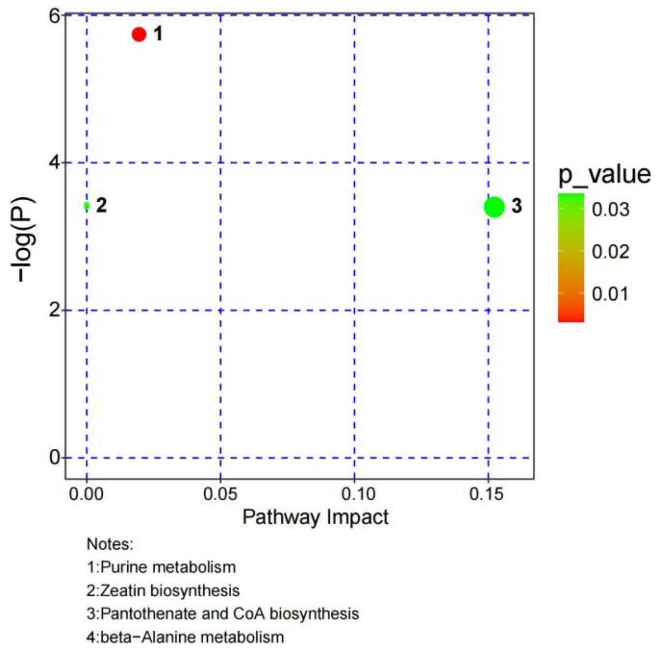

H

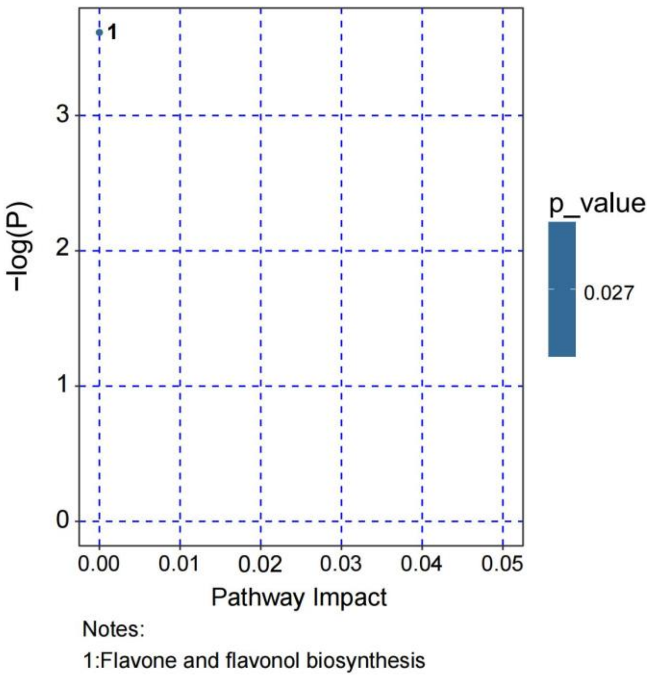

J

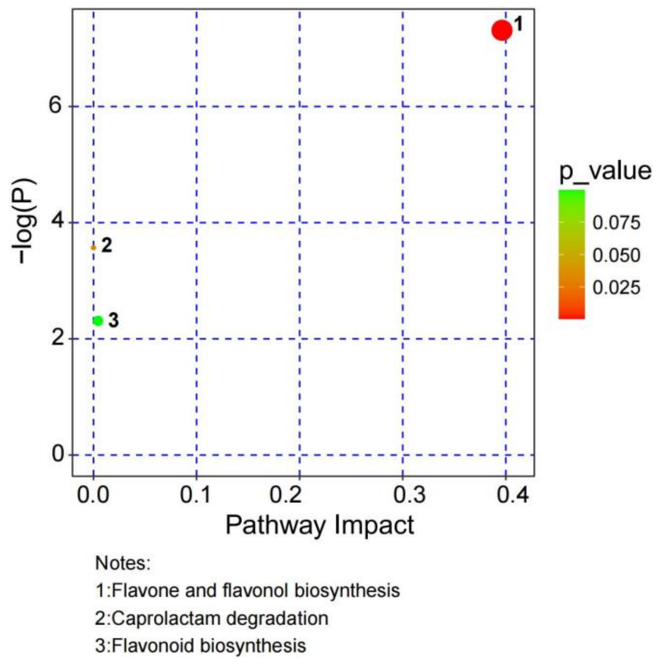

C

Fig. S5 Scatterplot of metabolites on the basis of KEGG.
