# Supplementary material for: Responses of transcriptome and metabolome in peanut leaves to dibutyl phthalate during whole growth period
Source: Front Plant Sci. 2024 Sep 20;15:1448971. doi: 10.3389/fpls.2024.1448971 (PMC11452913; doi:10.3389/fpls.2024.1448971)
Supplement: Supplementary file 1 [file DataSheet1.zip › Figure S7.PDF]

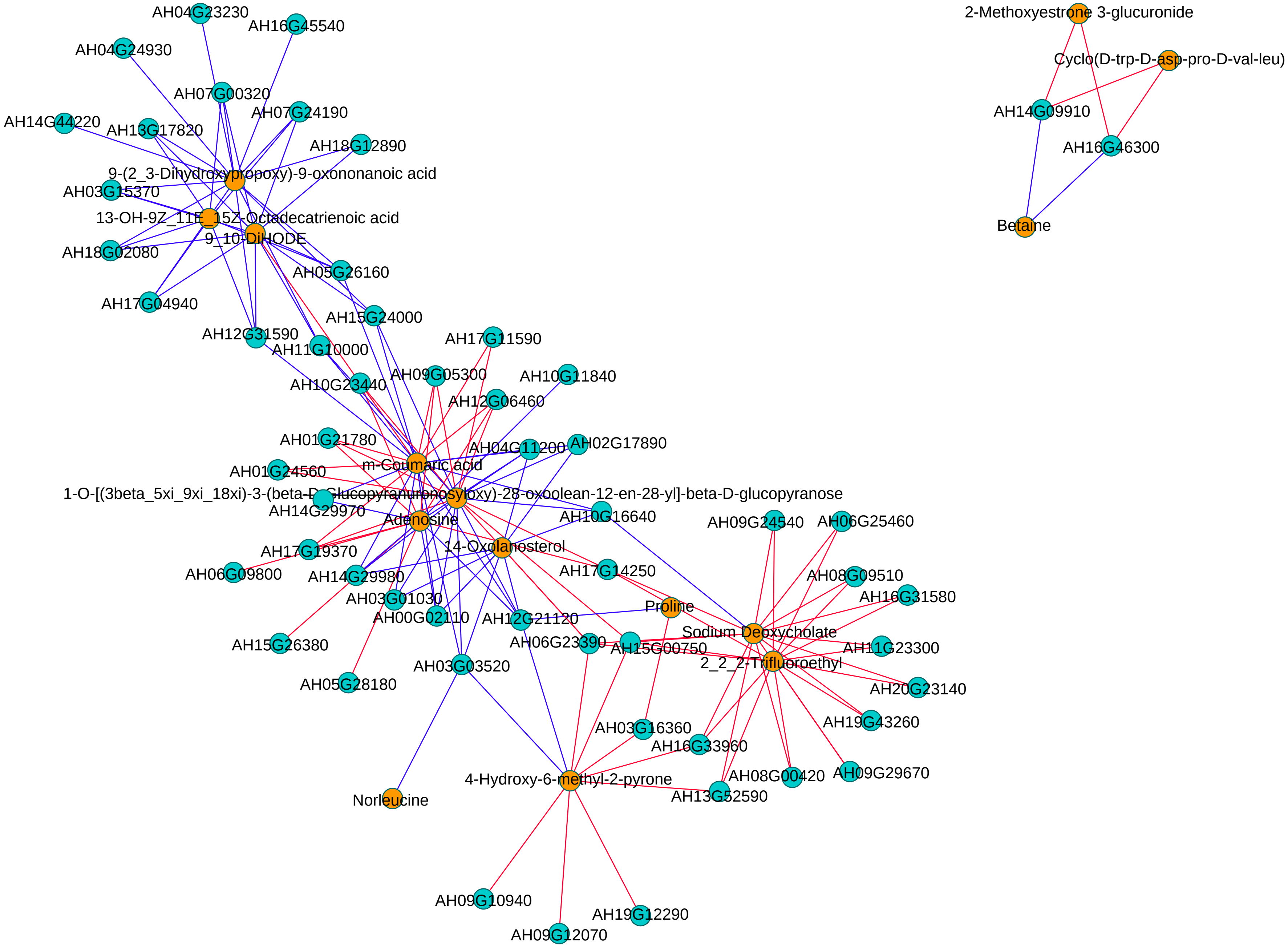
2-Methoxyestrone 3-glucuronide
Cyclo(D-trp-D-asp-pro-D-val-leu)
AH14G09910
AH16G46300
Betaine
AH04G23230
AH16G45540
AH04G24930
AH07G00320
AH07G24190
AH14G44220
AH13G17820
AH18G12890
9-(2_3-Dihydroxypropoxy)-9-oxononanoic acid
AH03G15370
13-OH-9Z_11E_15Z-Octadecatrienoic acid
9_10-DiHODE
AH18G02080
AH05G26160
AH17G04940
AH15G24000
AH12G31590
AH11G10000
AH17G11590
AH09G05300
AH10G11840
AH10G23440
AH12G06460
AH01G21780
AH04G11200
AH02G17890
m-Coumaric acid
AH01G24560
1-O-[(3beta_5xi_9xi_18xi)-3-(beta-D-Glucopyranuronosyloxy)-28-oxoolean-12-en-28-yl]-beta-D-glucopyranose
AH14G29970
Adenosine
AH10G16640
AH09G24540
AH06G25460
AH17G19370
14-Oxolanosterol
AH06G09800
AH14G29980
AH17G14250
AH08G09510
AH16G31580
AH03G01030
Proline
AH00G02110
AH12G21120
Sodium Deoxycholate
AH15G26380
AH06G23390
AH15G00750
AH11G23300
2_2_2-Trifluoroethyl
AH03G03520
AH05G28180
AH20G23140
AH19G43260
AH03G16360
AH16G33960
AH08G00420
AH09G29670
4-Hydroxy-6-methyl-2-pyrone
AH13G52590
Norleucine
AH09G10940
AH19G12290
AH09G12070

M

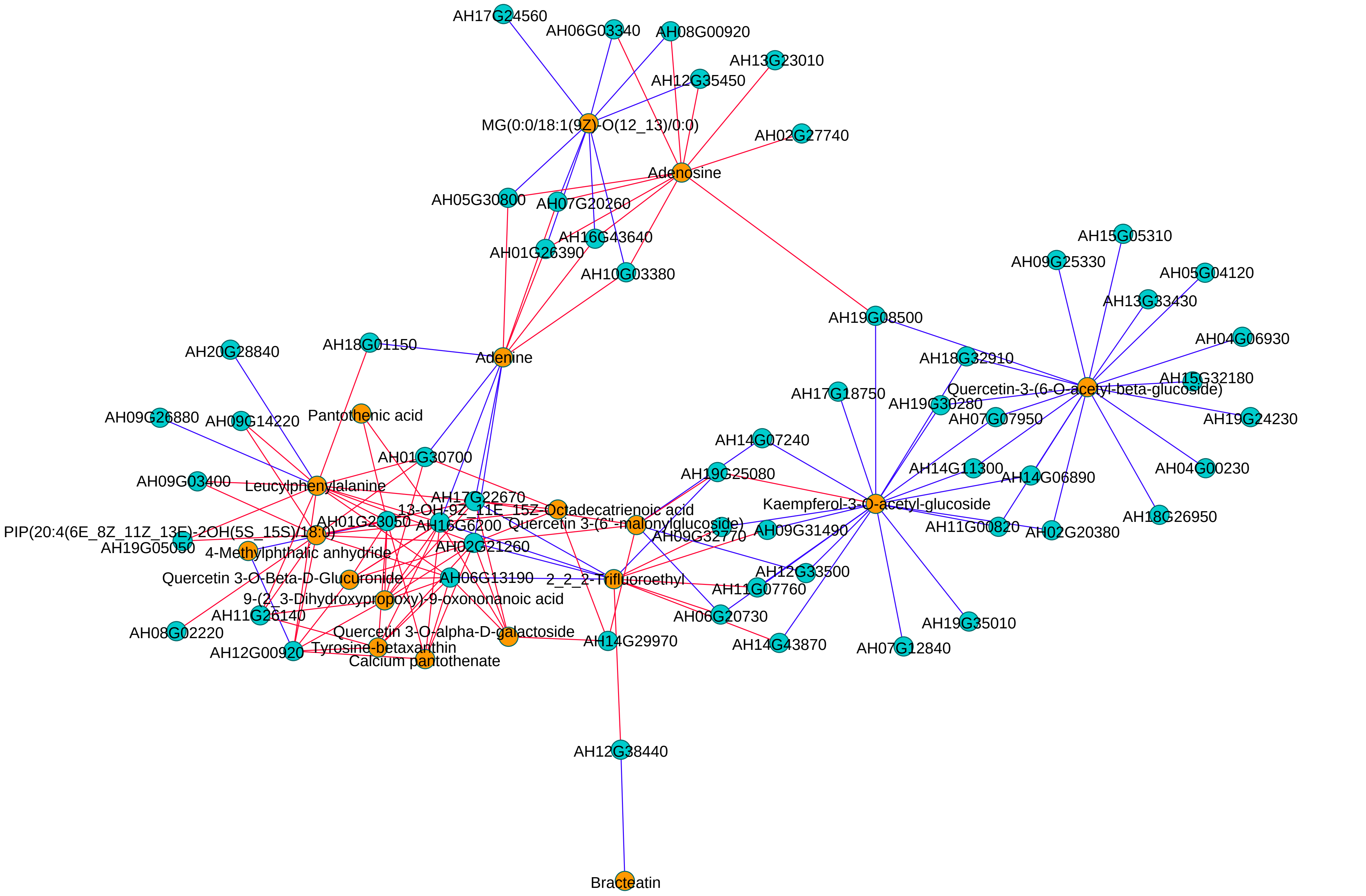
AH17G24560
AH06G03340
AH08G00920
AH13G23010
AH12G35450
MG(0:0/18:1(9Z)-O(12_13)/0:0)
AH02G27740
Adenosine
AH05G30800
AH07G20260
AH16G43640
AH01G26390
AH10G03380
AH15G05310
AH09G25330
AH05G04120
AH13G33430
AH19G08500
AH04G06930
AH20G28840
AH18G01150
Adenine
AH18G32910
AH15G32180
Quercetin-3-(6-O-acetyl-beta-glucoside)
AH17G18750
AH19G30280
AH09G26880
AH09G14220
Pantothenic acid
AH07G07950
AH19G24230
AH14G07240
AH01G30700
AH14G11300
AH14G06890
AH04G00230
AH09G03400
Leucylphenylalanine
AH19G25080
AH17G22670
Kaempferol-3-O-acetyl-glucoside
13-OH-9Z_11E_15Z-Octadecatrienoic acid
AH18G26950
AH01G23050
Quercetin 3-(6''-malonylglucoside)
AH16G6200
AH11G00820
AH02G20380
PIP(20:4(6E_8Z_11Z_13E)-2OH(5S_15S)/18:0)
AH09G32770
AH09G31490
AH19G05050
AH02G21260
4-Methylphthalic anhydride
AH12G33500
Quercetin 3-O-Beta-D-Glucuronide
AH06G13190
2_2_2-Trifluoroethyl
AH11G07760
9-(2_3-Dihydroxypropoxy)-9-oxononanoic acid
AH11G26140
AH06G20730
AH19G35010
AH08G02220
Quercetin 3-O-alpha-D-galactoside
AH14G29970
AH14G43870
AH07G12840
AH12G00920
Tyrosine-betaxanthin
Calcium pantothenate
AH12G38440
Bracteatin

H

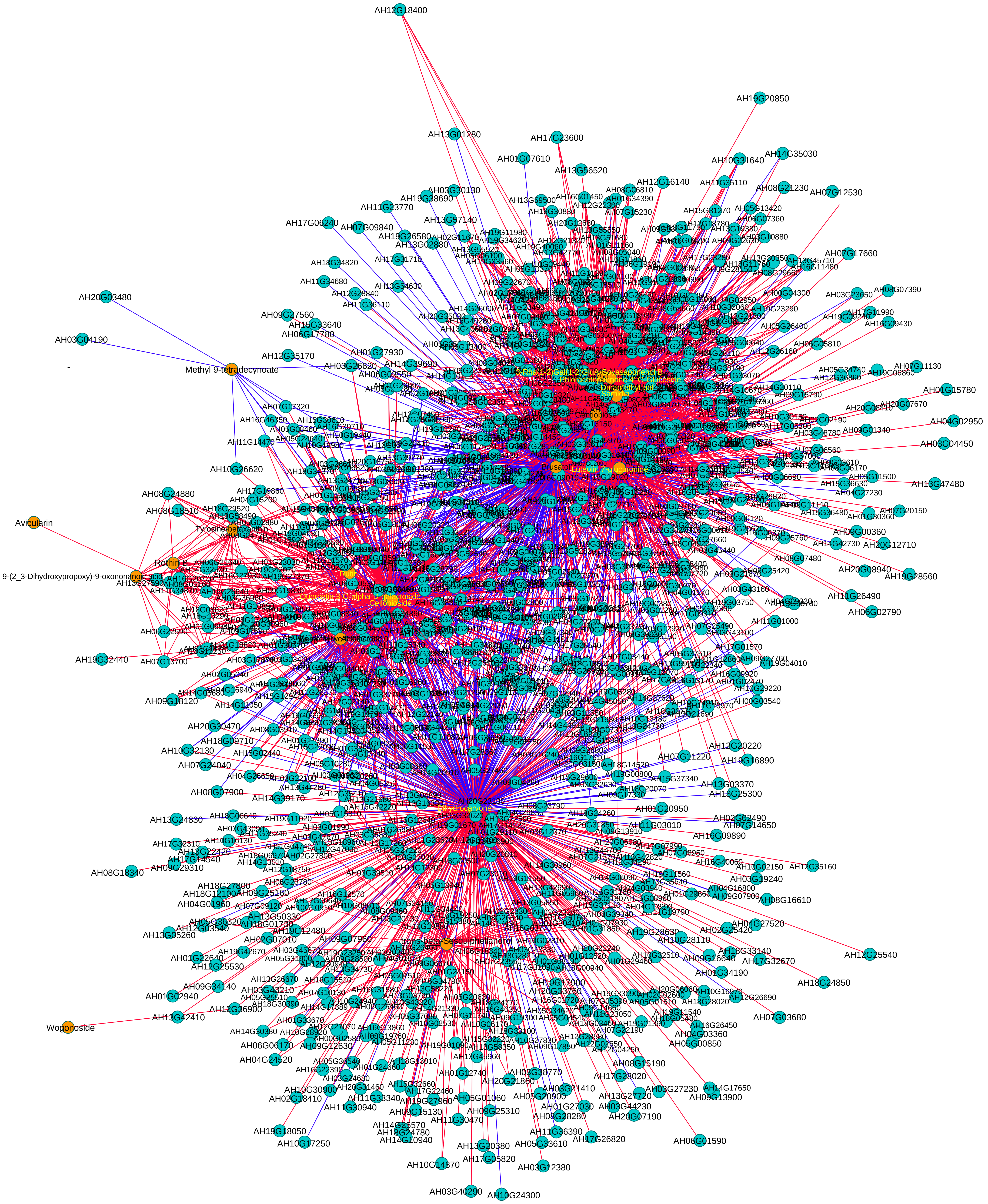

AH12G18400
AH19G20850
AH13G01280
AH17G23600
AH10G31640
AH14G35030
AH01G07610
AH13G56520
AH12G16140
AH08G21230
AH07G12530
AH03G30130
AH19G38690
AH11G23770
AH13G57140
AH17G06240
AH07G09840
AH19G26580
AH13G02880
AH07G17660
AH18G34820
AH17G31710
AH11G34680
AH13G54630
AH12G28840
AH11G36110
AH20G03480
AH09G27560
AH15G33640
AH06G17780
AH03G04190
Methyl 9-tetradecynoate
AH12G35170
AH01G27930
AH03G26620
AH14G39690
AH06G03550
AH07G17320
AH16G46350
AH11G14470
AH10G26620
AH08G24880
AH08G18510
Avicularin
AH18G29520
Tyrosine betaxanthin
Rothin B
9-(2_3-Dihydroxypropoxy)-9-oxononanoic acid
AH19G32440
AH07G13700
AH09G18120
AH20G30470
AH18G09710
AH10G32130
AH07G24040
AH08G07900
AH14G39170
AH13G24830
AH13G22420
AH17G14540
AH08G18340
AH09G29310
AH18G27800
AH18G12100
AH09G25160
AH04G01960
AH05G36320
AH12G03540
AH13G50330
AH13G05260
AH02G07010
AH19G12480
AH09G07960
AH01G22640
AH12G25530
AH09G34140
AH01G02940
AH12G36900
AH13G42410
Wogonoside
AH06G06170
AH04G24520
AH09G12630
AH10G30900
AH02G18410
AH11G30940
AH11G33340
AH19G18050
AH10G17250
AH09G15130
AH19G27960
AH14G25570
AH18G24780
AH14G10940
AH11G30470
AH05G01060
AH09G25310
AH10G14870
AH13G20380
AH17G05820
AH03G12380
AH03G40290
AH10G24300
AH05G33610
AH17G26820
AH11G36390
AH08G28280
AH01G27030
AH20G07190
AH03G44230
AH13G27720
AH03G27230
AH09G13900
AH14G17650
AH06G01590
AH17G28020
AH08G15190
AH03G38770
AH20G21860
AH03G21410
AH05G20900
trans-beta-Sesquiphellandrol
AH07G03680
AH18G24850
AH12G25540
AH17G32670
AH18G33140
AH01G34190
AH09G16640
AH10G28110
AH19G28630
AH02G25420
AH04G27520
AH03G19240
AH08G16610
AH12G35160
AH16G09890
AH07G14650
AH02G02490
AH01G20950
AH11G03010
AH13G03370
AH13G25300
AH19G16890
AH12G20220
AH07G11220
AH06G02790
AH11G26490
AH19G28560
AH20G08940
AH20G12710
AH09G00360
AH14G42730
AH13G47480
AH03G04450
AH04G02950
AH01G15780
AH07G11130
AH19G06860
AH07G20670
AH20G08410
AH16G09430
AH17G11990
AH03G23650
AH08G07390
AH07G20150
AH01G30360
Rutin
Quercetin 3-O-alpha-L-rhamnoside
Cellobioside
Busatol
Luteolin
Phloracetophenone 4'-O-Glucoside
Soyasapogenol B
Dimethylphenol
Sakuranetin
Vicenin

J

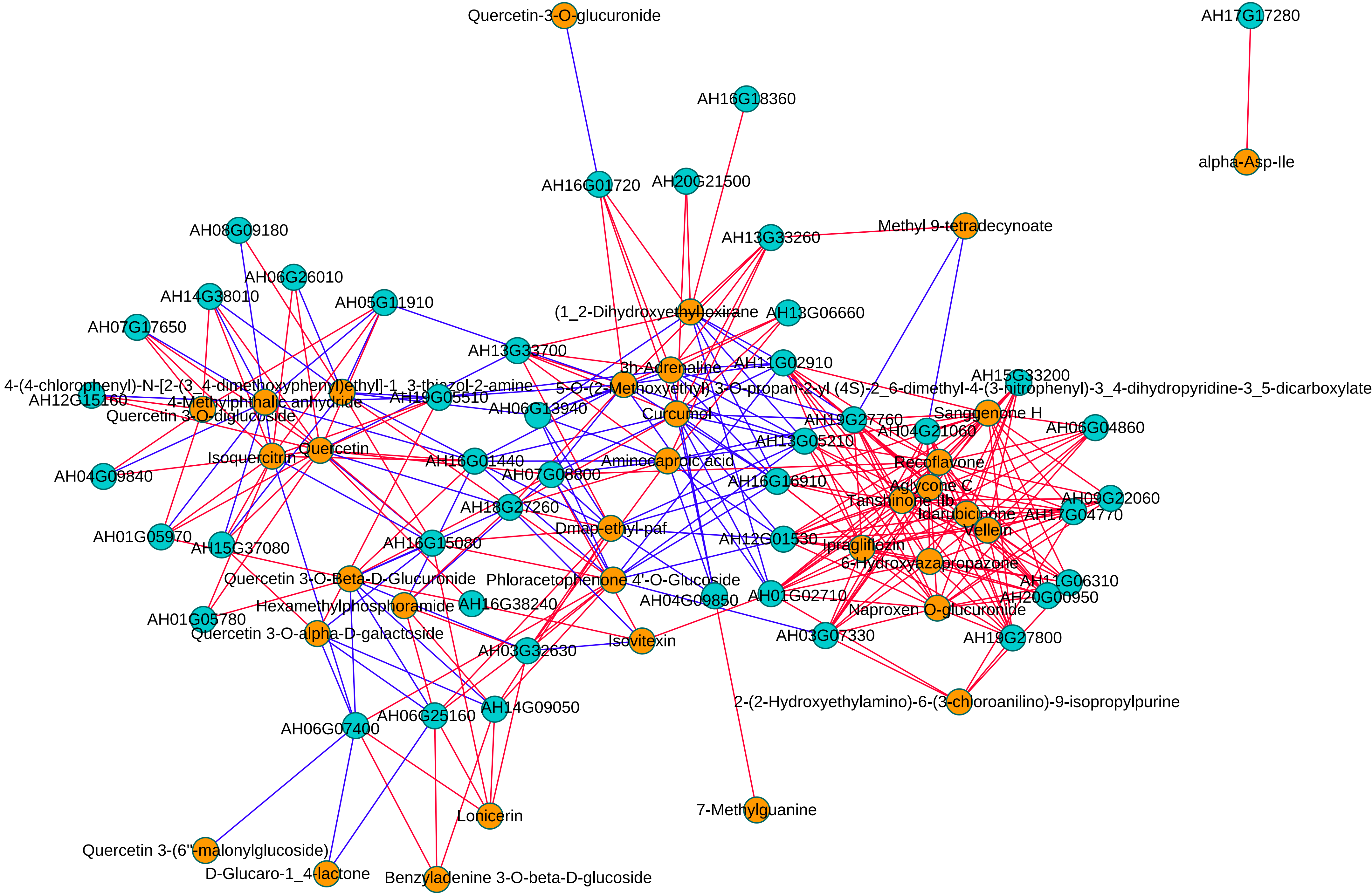
Quercetin-3-O-glucuronide
AH17G17280
alpha-Asp-Ile
AH16G18360
AH16G01720
AH20G21500
Methyl 9-tetradecynoate
AH13G33260
AH08G09180
AH06G26010
AH14G38010
AH05G11910
(1_2-Dihydroxyethyl)oxirane
AH13G06660
AH07G17650
AH13G33700
3h-Adrenaline
AH11G02910
AH15G33200
4-(4-chlorophenyl)-N-[2-(3_4-dimethoxyphenyl)ethyl]-1_3-thiazol-2-amine
5-O-(2-Methoxyethyl) 3-O-propan-2-yl (4S)-2_6-dimethyl-4-(3-nitrophenyl)-3_4-dihydropyridine-3_5-dicarboxylate
AH12G15160
4-Methylphthalic anhydride
AH19G05510
AH06G13940
Quercetin 3-O-diglucoside
Curcumol
Sanggenone H
AH19G27760
AH04G21060
AH06G04860
AH13G05210
Isoquercitrin
Quercetin
AH16G01440
Aminocaproic acid
Recoflavone
AH04G09840
AH07G08800
AH16G16910
Aglycone C
Tanshinone IIb
AH09G22060
AH18G27260
Idarubicinone
AH17G04770
Dmap-ethyl-paf
Vellein
AH01G05970
AH15G37080
AH16G15080
AH12G01530
Ipragliflozin
6-Hydroxyazapropazone
Quercetin 3-O-Beta-D-Glucuronide
Phloracetophenone 4'-O-Glucoside
AH11G06310
AH01G02710
AH20G00950
AH04G09850
Hexamethylphosphoramide
AH16G38240
Naproxen O-glucuronide
AH01G05780
Quercetin 3-O-alpha-D-galactoside
AH03G32630
Isovitexin
AH03G07330
AH19G27800
2-(2-Hydroxyethylamino)-6-(3-chloroanilino)-9-isopropylpurine
AH06G25160
AH14G09050
AH06G07400
Lonicerin
7-Methylguanine
Quercetin 3-(6''-malonylglucoside)
D-Glucaro-1_4-lactone
Benzyladenine 3-O-beta-D-glucoside

C

Fig.S7 The inferred networks of metabolites and transcript variables.Yellow circles representing metabolites and blue circles representing genes; Each line represents an interaction between the gene and/or metabolite, with the red line representing a positive correlation and the blue line representing a negative correlation.
