# Supplementary material for: Responses of transcriptome and metabolome in peanut leaves to dibutyl phthalate during whole growth period
Source: Front Plant Sci. 2024 Sep 20;15:1448971. doi: 10.3389/fpls.2024.1448971 (PMC11452913; doi:10.3389/fpls.2024.1448971)
Supplement: Supplementary file 1 [file DataSheet1.zip › Supplemental Table S1 - S2.DOCX]

**Supplemental Table S1** Quality control of transcriptome data

| **Sample** | **Raw_reads_num** | **Raw_bases**  **(G)** | **Clean_reads_num** | **Clean_bases**  **(G)** | **Clean_rate**  **(%)** | **Q20(%)** | **Q30(%)** | **GC(%)** | **RIN** |
| --- | --- | --- | --- | --- | --- | --- | --- | --- | --- |
| M_CK_S1 | 41,251,722 | 6.19 | 41,250,252 | 6.17 | 100 | 98.98 | 95.87 | 45.69 | 7.75 |
| M_CK_S2 | 58,521,330 | 8.78 | 58,520,614 | 8.65 | 100 | 99.12 | 96.89 | 45.41 | 7.60 |
| M_CK_S3 | 79,443,246 | 11.92 | 79,442,166 | 11.88 | 100 | 98.36 | 94.71 | 44.83 | 9.71 |
| M_DBP_S1 | 49,468,092 | 7.42 | 49,467,372 | 7.40 | 100 | 98.50 | 95.11 | 46.22 | 9.24 |
| M_DBP_S2 | 48,327,750 | 7.25 | 48,325,852 | 7.23 | 100 | 99.13 | 96.44 | 45.86 | 9.73 |
| M_DBP_S3 | 43,413,902 | 6.51 | 43,413,108 | 6.49 | 100 | 98.76 | 95.79 | 46.57 | 8.72 |
| H_CK_S1 | 62,983,262 | 9.45 | 62,980,716 | 9.42 | 100 | 99.24 | 96.89 | 45.84 | 8.60 |
| H_CK_S2 | 122,281,098 | 18.34 | 122,276,150 | 18.12 | 100 | 99.03 | 96.62 | 45.81 | 8.84 |
| H_CK_S3 | 49,962,600 | 7.49 | 49,960,312 | 7.47 | 100 | 98.70 | 95.68 | 45.63 | 8.66 |
| H_DBP_S1 | 89,065,630 | 13.36 | 89,054,248 | 13.32 | 99.99 | 98.73 | 95.18 | 45.76 | 9.02 |
| H_DBP_S2 | 70,948,408 | 10.64 | 70,943,644 | 10.62 | 99.99 | 98.39 | 94.83 | 45.38 | 9.16 |
| H_DBP_S3 | 79,255,418 | 11.89 | 79,249,220 | 11.85 | 99.99 | 99.15 | 96.55 | 45.11 | 8.64 |
| J_CK_S1 | 46,263,588 | 6.94 | 46,262,966 | 6.92 | 100 | 98.35 | 94.67 | 46.8 | 8.58 |
| J_CK_S2 | 68,386,556 | 10.26 | 68,385,792 | 10.24 | 100 | 98.28 | 94.37 | 46.95 | 8.52 |
| J_CK_S3 | 51,094,502 | 7.66 | 51,093,730 | 7.65 | 100 | 98.29 | 94.54 | 47.48 | 9.28 |
| J_DBP_S1 | 38,057,474 | 5.71 | 38,055,756 | 5.70 | 100 | 99.16 | 96.54 | 46.86 | 9.78 |
| J_DBP_S2 | 69,306,686 | 10.4 | 69,305,148 | 10.38 | 100 | 98.17 | 94.12 | 48.10 | 9.49 |
| J_DBP_S3 | 43,076,154 | 6.46 | 43,074,392 | 6.45 | 100 | 99.17 | 96.67 | 47.09 | 9.64 |
| C_CK_S1 | 98,407,326 | 14.76 | 98,404,948 | 14.75 | 100 | 97.76 | 92.82 | 53.41 | 9.65 |
| C_CK_S2 | 126,189,880 | 18.93 | 126,187,214 | 18.89 | 100 | 98.21 | 94.30 | 48.63 | 8.57 |
| C_CK_S3 | 101,743,790 | 15.26 | 101,742,254 | 15.22 | 100 | 99.34 | 97.31 | 45.86 | 9.32 |
| C_DBP_S1 | 62,706,702 | 9.41 | 62,704,158 | 9.39 | 100 | 98.31 | 94.52 | 46.17 | 9.71 |
| C_DBP_S2 | 52,968,628 | 7.95 | 52,966,230 | 7.93 | 100 | 99.25 | 96.99 | 46.29 | 8.99 |
| C_DBP_S3 | 51,826,868 | 7.77 | 51,826,048 | 7.76 | 100 | 97.78 | 92.98 | 44.58 | 8.99 |

**Supplemental Table S2** Quality control of transcriptome data

| Seeding stage | P | N |
| --- | --- | --- |
| 2_2_2-Trifluoroethyl | 14 | 0 |
| Sodium Deoxycholate | 14 | 1 |
| 1-O-[(3beta_5xi_9xi_18xi)-3-(beta-D-Glucopyranuronosyloxy)-28-oxoolean-12-en-28-yl]-beta-D-glucopyranose | 10 | 11 |
| Adenosine | 9 | 6 |
| 4-Hydroxy-6-methyl-2-pyrone | 8 | 2 |
| m-Coumaric acid | 7 | 13 |
| 2-Methoxyestrone 3-glucuronide | 2 | 0 |
| Cyclo(D-trp-D-asp-pro-D-val-leu) | 2 | 0 |
| 9_10-DiHODE | 1 | 11 |
| 14-Oxolanosterol | 1 | 8 |
| Proline | 1 | 1 |
| 13-OH-9Z_11E_15Z-Octadecatrienoic acid | 0 | 8 |
| 9-(2_3-Dihydroxypropoxy)-9-oxononanoic acid | 0 | 15 |
| Betaine | 0 | 2 |
| Norleucine | 0 | 1 |
| Flowering stage | P | N |
| Leucylphenylalanine | 12 | 2 |
| PIP(20:4(6E_8Z_11Z_13E)-2OH(5S_15S)/18:0) | 12 | 0 |
| Adenosine | 11 | 0 |
| Quercetin 3-O-Beta-D-Glucuronide | 11 | 0 |
| 9-(2_3-Dihydroxypropoxy)-9-oxononanoic acid | 8 | 0 |
| 2_2_2-Trifluoroethyl | 6 | 5 |
| Tyrosine-betaxanthin | 6 | 0 |
| 13-OH-9Z_11E_15Z-Octadecatrienoic acid | 5 | 0 |
| Adenine | 5 | 5 |
| Calcium pantothenate | 5 | 0 |
| Quercetin 3-(6''-malonylglucoside) | 4 | 3 |
| Pantothenic acid | 2 | 0 |
| Kaempferol-3-O-acetyl-glucoside | 1 | 18 |
| 4-Methylphthalic anhydride | 0 | 2 |
| Bracteatin | 0 | 1 |
| MG(0:0/18:1(9Z)-O(12_13)/0:0) | 0 | 9 |
| Quercetin-3-(6-O-acetyl-beta-glucoside) | 0 | 17 |
| Pod stage | P | N |
| Brusatol | 366 | 180 |
| Dihydrocarvone | 329 | 211 |
| Samidin | 287 | 14 |
| Gentiobiose | 271 | 36 |
| 3-Rha(1-2)Gal(1-2)GluA-Soyasaponenol B | 258 | 0 |
| Cellobioside | 212 | 62 |
| Quercetin 3-O-alpha-D-galactoside | 206 | 56 |
| Phloracetophenone 4'-O-Glucoside | 178 | 0 |
| Demethylwedelolactone | 119 | 21 |
| Luteolin 7-glucuronide | 110 | 305 |
| trans-beta-Sesquiphellandrol | 85 | 1 |
| Dmap-ethyl-paf | 84 | 0 |
| Rutin | 42 | 0 |
| Tyrosine-betaxanthin | 27 | 0 |
| Rothin B | 26 | 0 |
| 9-(2_3-Dihydroxypropoxy)-9-oxononanoic acid | 18 | 0 |
| Avicularin | 1 | 0 |
| Wogonoside | 1 | 0 |
| Methyl 9-tetradecynoate | 0 | 11 |
| Maturity stage | P | N |
| Recoflavone | 16 | 0 |
| Ipragliflozin | 15 | 0 |
| Naproxen O-glucuronide | 15 | 0 |
| Sanggenone H | 15 | 0 |
| Tanshinone IIb | 15 | 0 |
| 6-Hydroxyazapropazone | 13 | 0 |
| Quercetin | 13 | 3 |
| Aglycone C | 11 | 0 |
| Idarubicinone | 11 | 0 |
| Vellein | 10 | 0 |
| Isoquercitrin | 9 | 3 |
| (1_2-Dihydroxyethyl)oxirane | 6 | 6 |
| Curcumol | 6 | 10 |
| Dmap-ethyl-paf | 6 | 7 |
| Phloracetophenone 4'-O-Glucoside | 6 | 8 |
| Quercetin-3-O-glucuronide | 6 | 1 |
| 2-(2-Hydroxyethylamino)-6-(3-chloroanilino)-9-isopropylpurine | 5 | 0 |
| 5-O-(2-Methoxyethyl) 3-O-propan-2-yl (4S)-2_6-dimethyl-4-(3-nitrophenyl)-3_4-dihydropyridine-3_5-dicarboxylate | 5 | 9 |
| Aminocaproic acid | 5 | 7 |
| Lonicerin | 5 | 0 |
| Quercetin 3-O-Beta-D-Glucuronide | 5 | 6 |
| 3h-Adrenaline | 4 | 7 |
| Hexamethylphosphoramide | 4 | 2 |
| 4-(4-chlorophenyl)-N-[2-(3_4-dimethoxyphenyl)ethyl]-1_3-thiazol-2-amine | 3 | 7 |
| Benzyladenine 3-O-beta-D-glucoside | 3 | 0 |
| Isovitexin | 3 | 2 |
| Quercetin 3-O-alpha-D-galactoside | 2 | 4 |
| 4-Methylphthalic anhydride | 1 | 9 |
| 7-Methylguanine | 1 | 0 |
| alpha-Asp-Ile | 1 | 0 |
| Methyl 9-tetradecynoate | 1 | 2 |
| D-Glucaro-1_4-lactone | 0 | 2 |
| Quercetin 3-(6''-malonylglucoside) | 0 | 1 |
