# Supplementary figures and images for: Responses of transcriptome and metabolome in peanut leaves to dibutyl phthalate during whole growth period
Source: Front Plant Sci. 2024 Sep 20;15:1448971. doi: 10.3389/fpls.2024.1448971 (PMC11452913; doi:10.3389/fpls.2024.1448971)

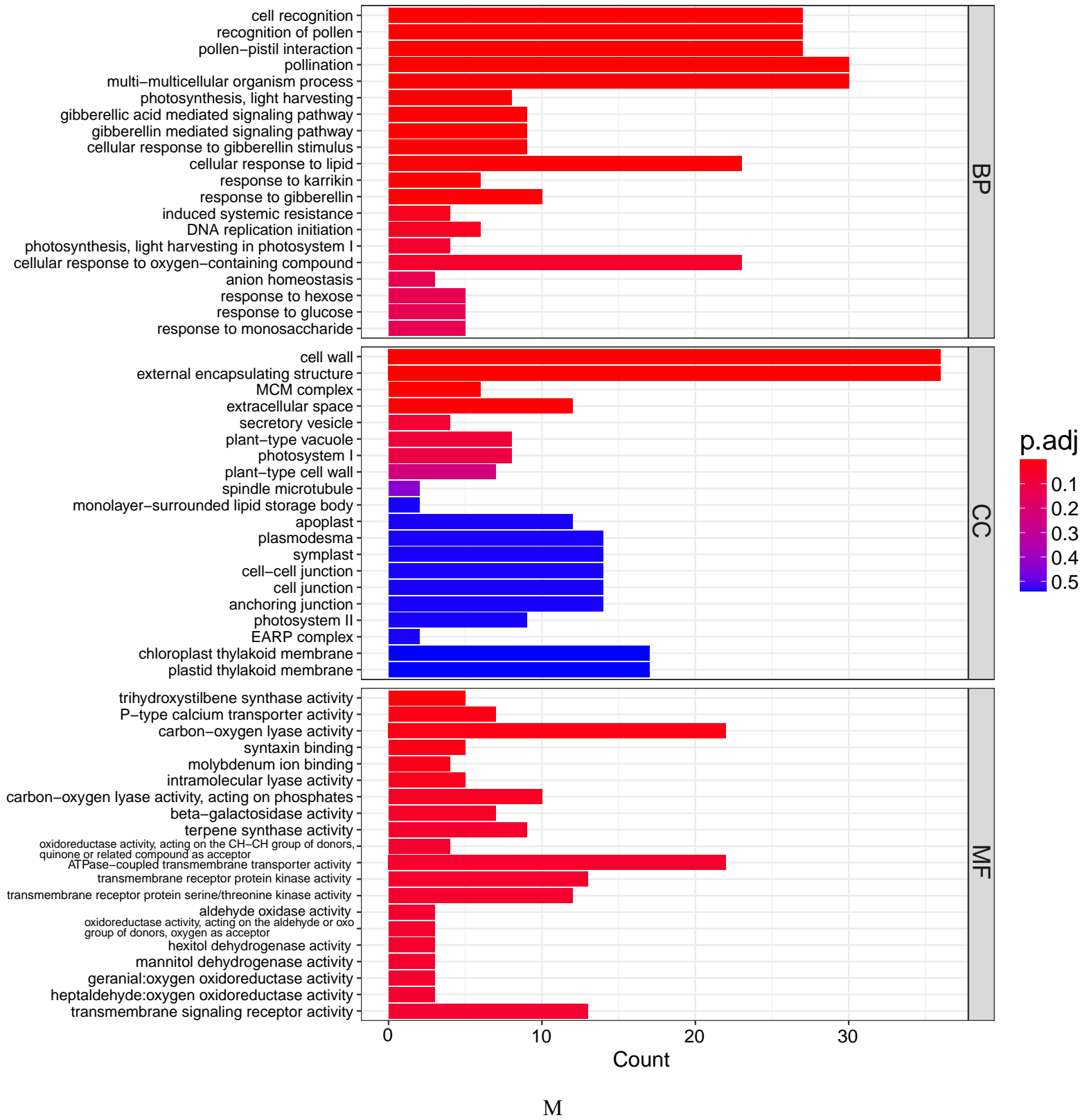

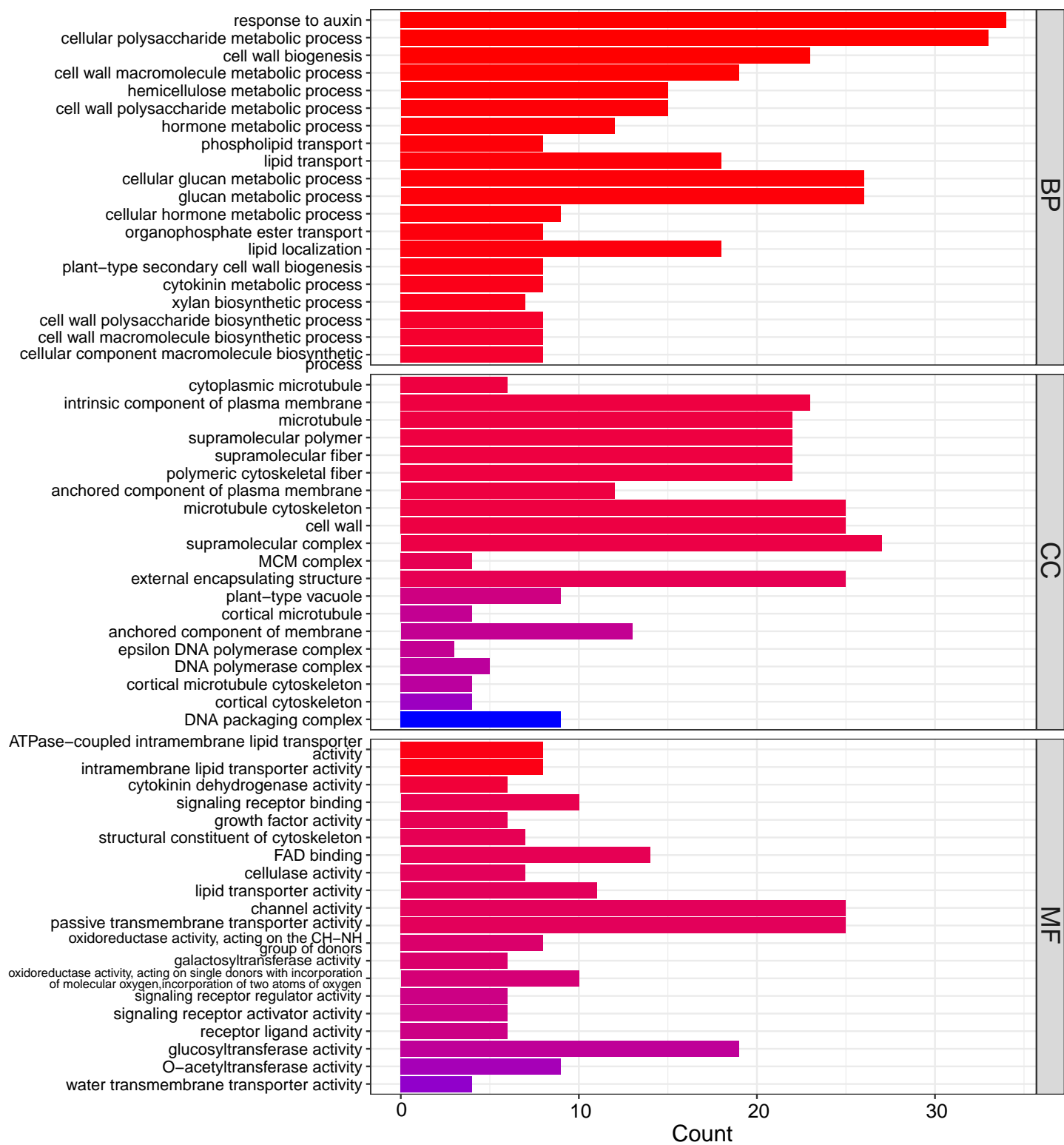

H

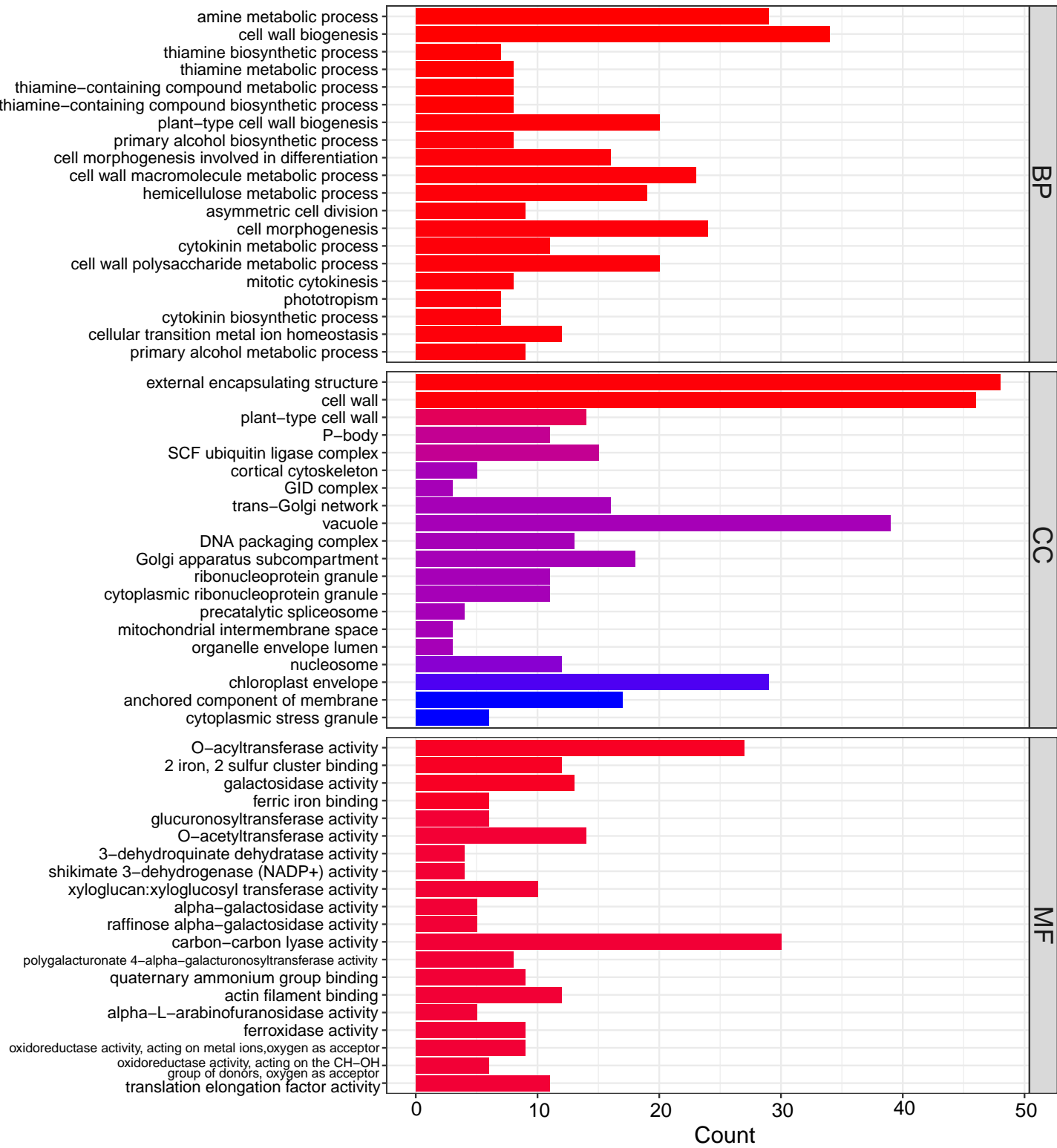

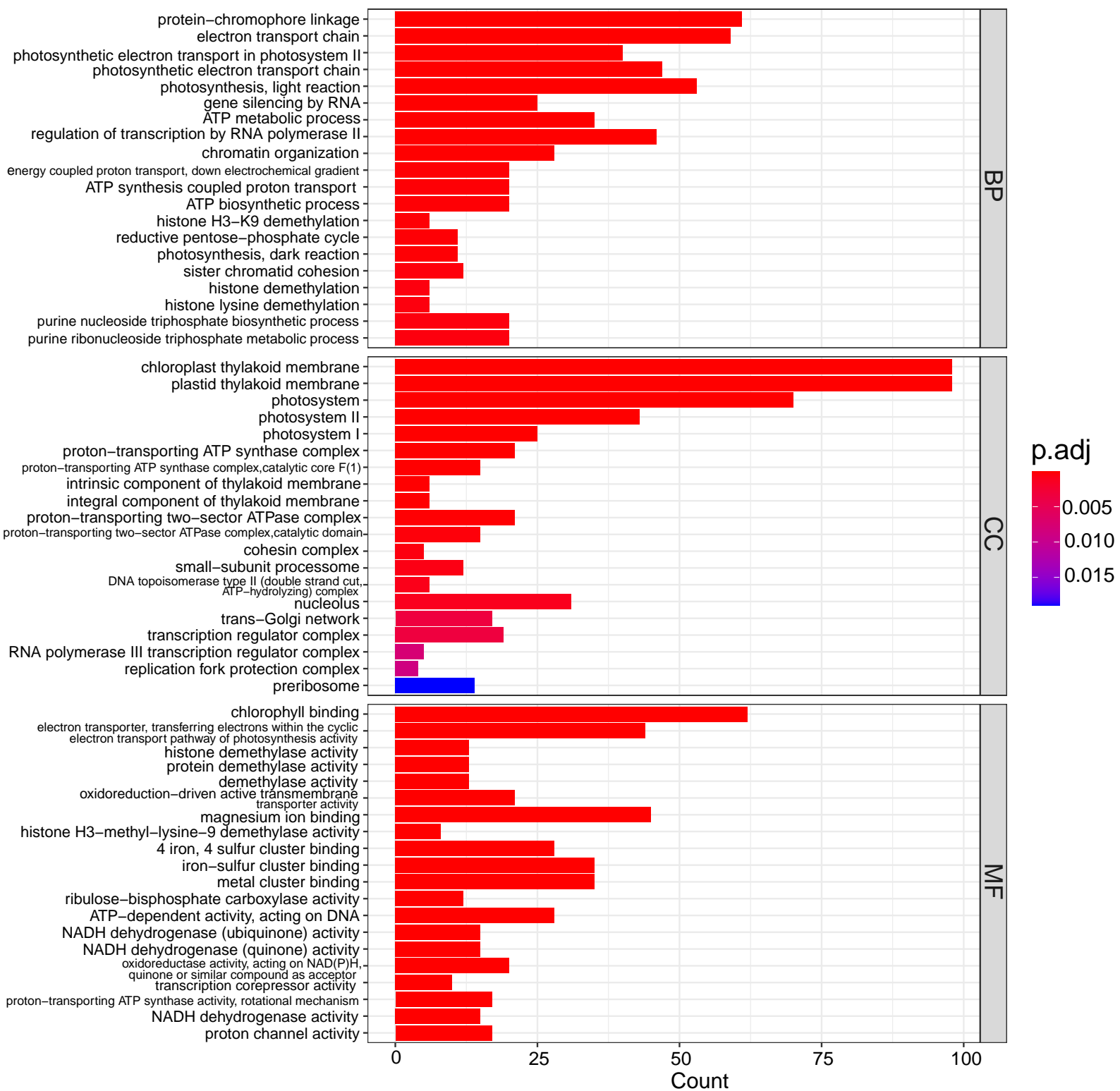

C

Fig. S1. GO annotation analysis.

Supplement: Supplementary file 1 [file DataSheet1.zip › Figure S1.PDF]

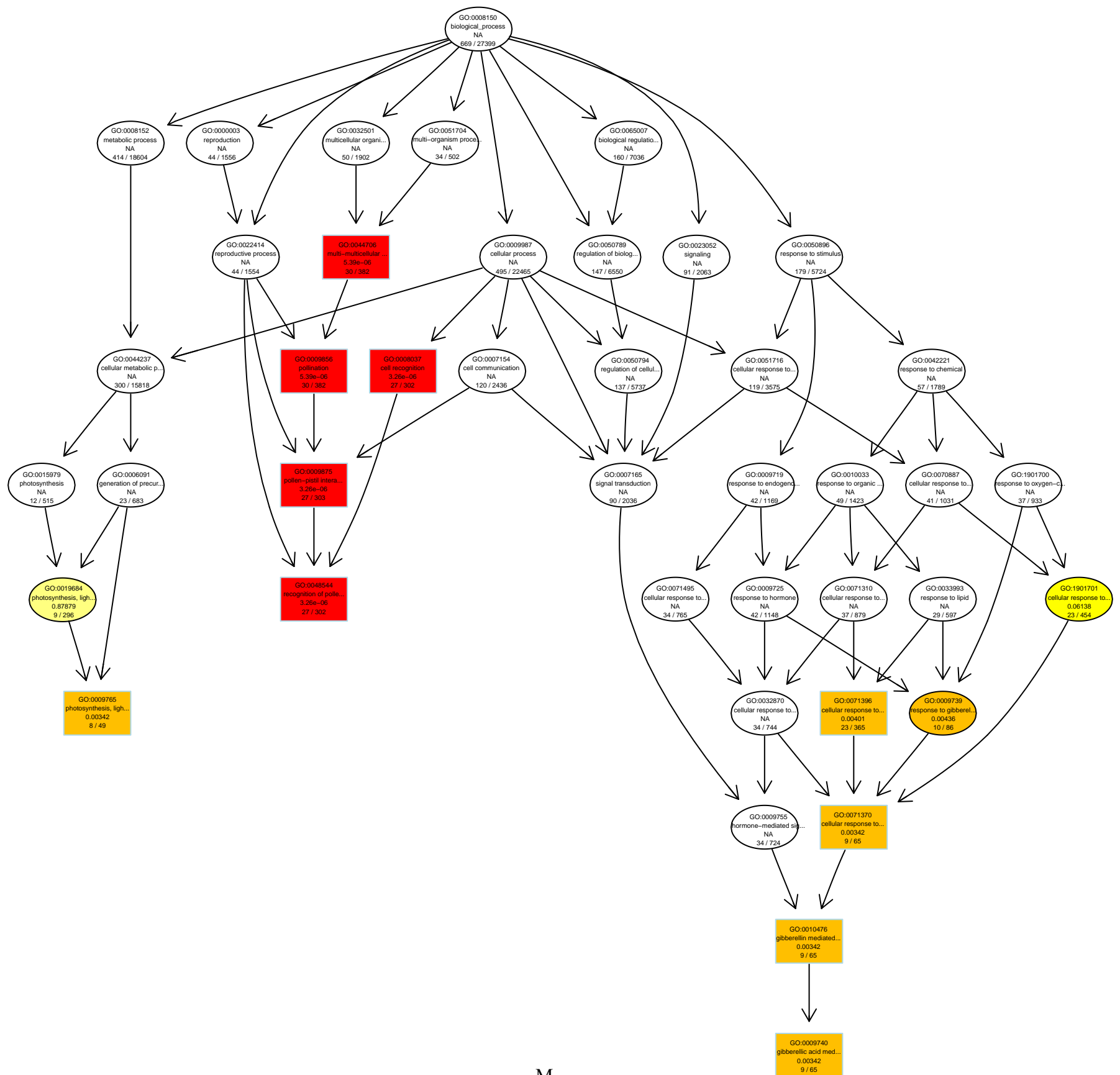

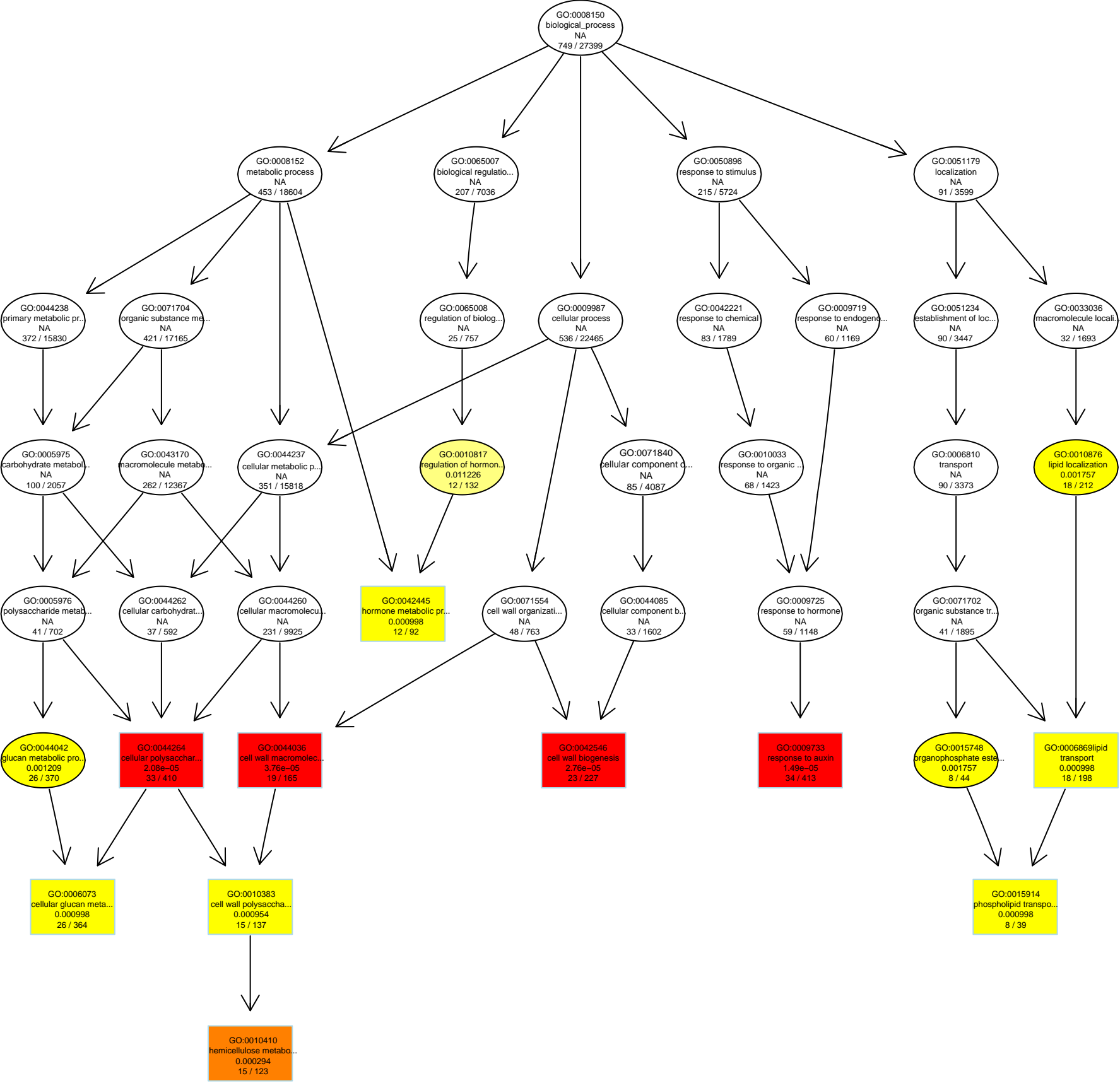

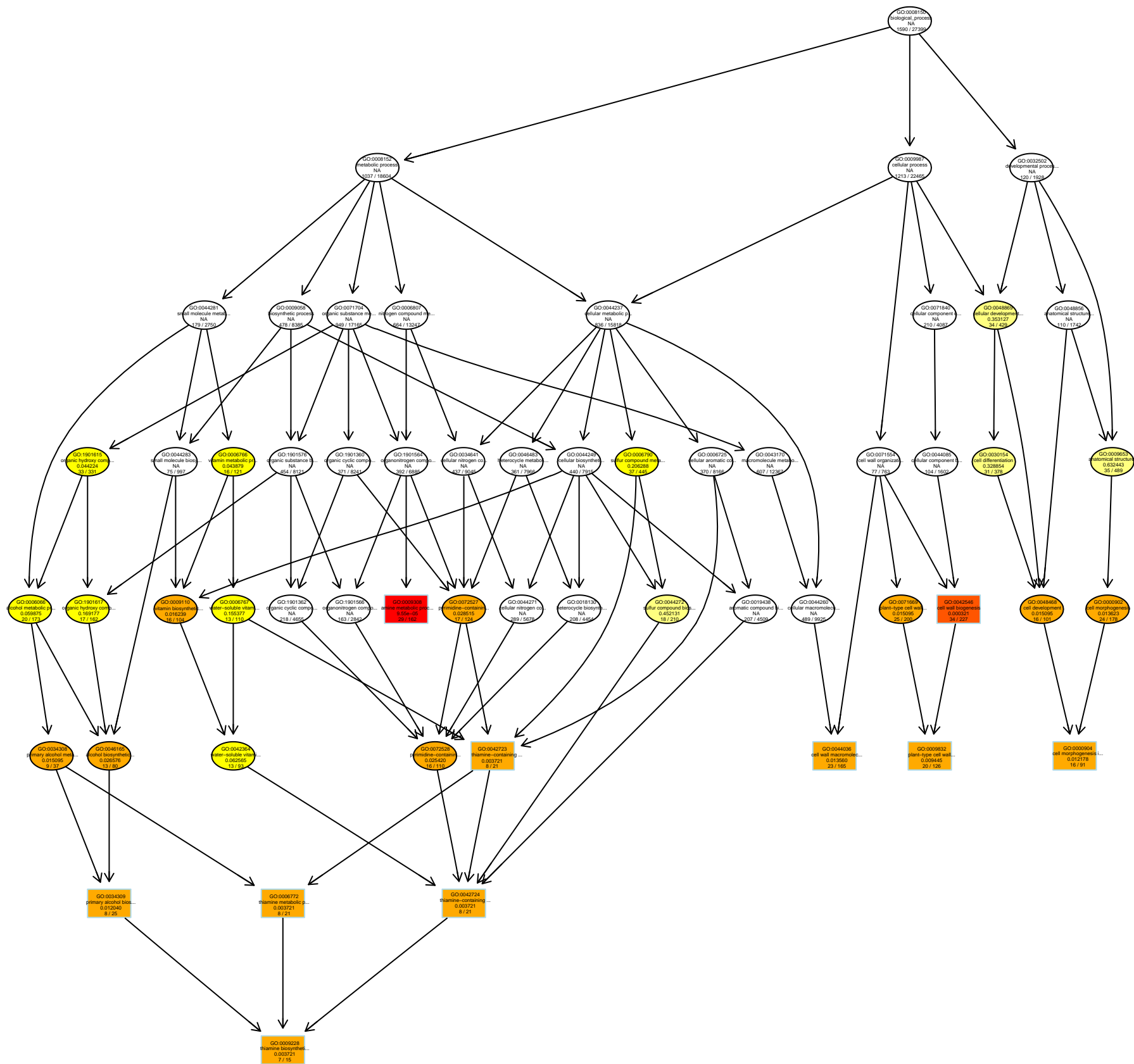

Supplement: Supplementary file 1 [file DataSheet1.zip › Figure S2.PDF]

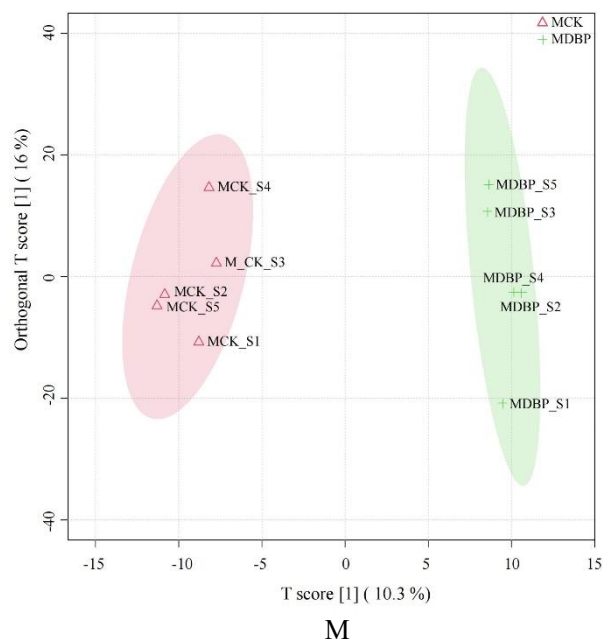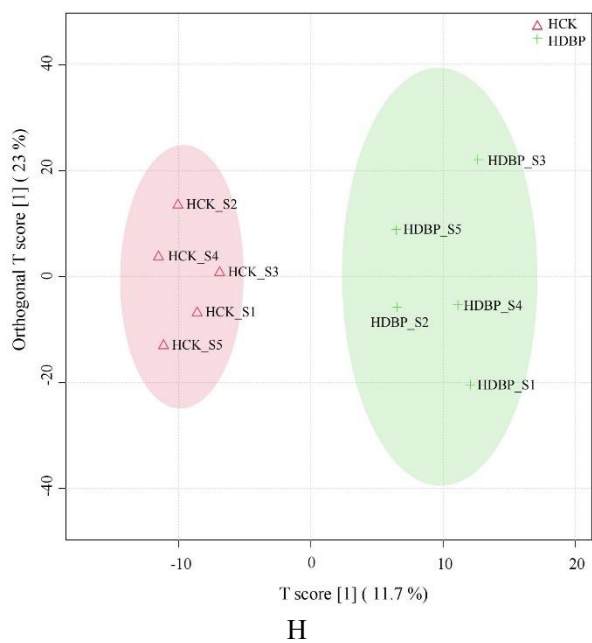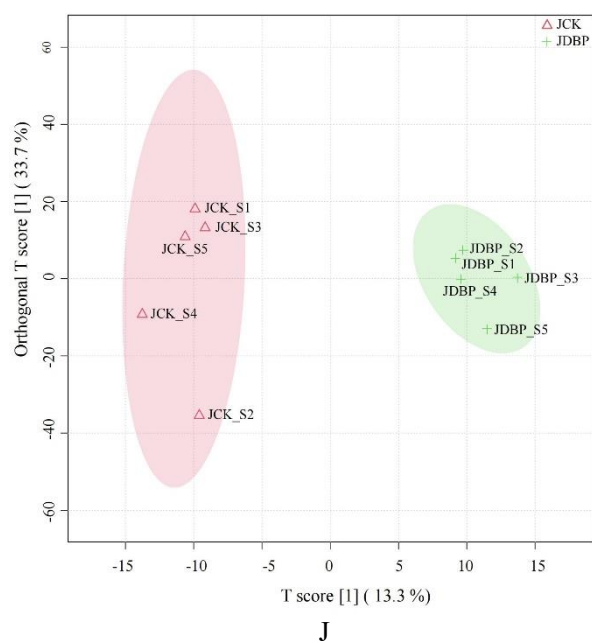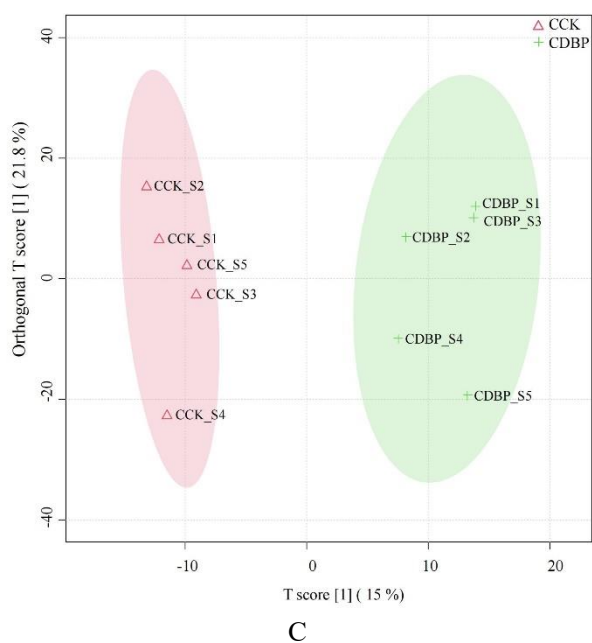

**Fig. S3** OPLS-DA scores plot.

Supplement: Supplementary file 1 [file DataSheet1.zip › Figure S3.PDF]

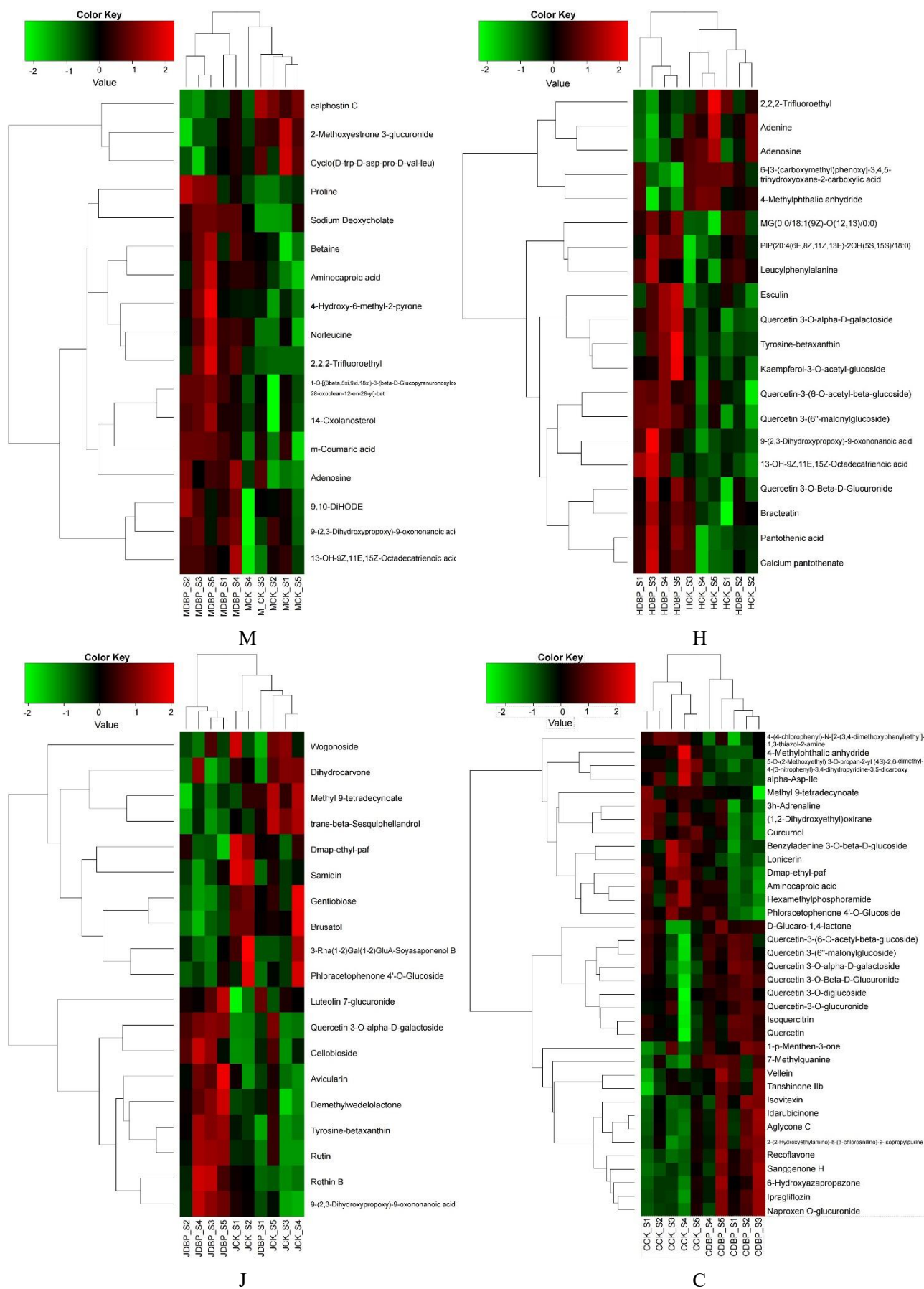

**Fig. S4** Heatmap showed the results of the clustering analysis of DAMs.

Supplement: Supplementary file 1 [file DataSheet1.zip › Figure S4.PDF]
